# Assessing the contribution of tumor mutational phenotypes to cancer progression risk

**DOI:** 10.1371/journal.pcbi.1008777

**Published:** 2021-03-12

**Authors:** Yifeng Tao, Ashok Rajaraman, Xiaoyue Cui, Ziyi Cui, Haoran Chen, Yuanqi Zhao, Jesse Eaton, Hannah Kim, Jian Ma, Russell Schwartz

**Affiliations:** 1 Computational Biology Department, School of Computer Science, Carnegie Mellon University, Pittsburgh, Pennsylvania, United States of America; 2 Joint Carnegie Mellon-University of Pittsburgh Ph.D. Program in Computational Biology, Pittsburgh, Pennsylvania, United States of America; 3 Department of Biological Sciences, Mellon College of Science, Carnegie Mellon University, Pittsburgh, Pennsylvania, United States of America; Max Delbruck Centrum fur Molekulare Medizin Berlin Buch, GERMANY

## Abstract

Cancer occurs via an accumulation of somatic genomic alterations in a process of clonal evolution. There has been intensive study of potential causal mutations driving cancer development and progression. However, much recent evidence suggests that tumor evolution is normally driven by a variety of mechanisms of somatic hypermutability, which act in different combinations or degrees in different cancers. These variations in mutability phenotypes are predictive of progression outcomes independent of the specific mutations they have produced to date. Here we explore the question of how and to what degree these differences in mutational phenotypes act in a cancer to predict its future progression. We develop a computational paradigm using evolutionary tree inference (tumor phylogeny) algorithms to derive features quantifying single-tumor mutational phenotypes, followed by a machine learning framework to identify key features predictive of progression. Analyses of breast invasive carcinoma and lung carcinoma demonstrate that a large fraction of the risk of future clinical outcomes of cancer progression—overall survival and disease-free survival—can be explained solely from mutational phenotype features derived from the phylogenetic analysis. We further show that mutational phenotypes have additional predictive power even after accounting for traditional clinical and driver gene-centric genomic predictors of progression. These results confirm the importance of mutational phenotypes in contributing to cancer progression risk and suggest strategies for enhancing the predictive power of conventional clinical data or driver-centric biomarkers.

## Author summary

Cancer results from mutations in previously healthy cell populations that typically accumulate over a period of years before tumor growth is apparent. This process is governed by the laws of evolution, by which random mutations arising in cells will occasionally lead to selection for particular cell populations with growth advantages. How this process unfolds in any given tumor is highly random and idiosyncratic, however, making tumor growth difficult to predict. One reason tumor evolution is so random is that tumors frequently contain mutations that bias the cancer cells to generate new mutations in different ways, yet variations among these mechanisms of mutability (which we call “mutational phenotypes”) themselves provide predictive power for a cancer’s future progression. However, little is known about the degree to which the risk of cancer progressing is characterized by these variations in mutation mechanism as opposed to other sources. In this work, we examine the question of how much of the risk of cancer progression is explained by these differences patient-to-patient in mutability preferences. We find that approximately a third of the risk of future cancer progression is accounted for by variations in mutational phenotypes. Furthermore, these mutational phenotypes are complementary to and only partially redundant with other sources of predictive information, a finding confirmed by showing that machine learning models using such information can enhance our ability to predict which cancers progress and recur beyond what can be accomplished from more traditional genomic and clinical data sources alone.

## Introduction

Cancers are typically caused by somatic genomic alterations accumulating under the forces of evolutionary diversification and selection that ultimately lead to uncontrolled cell growth [1]. In most cases, cancer progression is accelerated by somatic hypermutability, where defects in DNA replication or repair mechanisms cause the rapid acquisition of mutations across generations of cell growth [2]. Tumor cell populations thus typically undergo substantial genetic diversification over time, most of it likely selectively neutral but some with phenotypic effects [3], resulting in profound intra-tumor heterogeneity (ITH) [4], i.e., cell-to-cell variation in genetic makeup. Such heterogeneity in turn creates an opportunity for selection for mutations that promote uncontrolled cell growth or escape from normal controls on growth, leading ultimately to tumor development and potentially subsequent metastasis and patient mortality [1, 5]. This process of evolutionary “diversification” and “selection” further underlies the development of cancer recurrence and resistance to therapeutics [6]. Understanding the processes of somatic evolution that act in cancers is thus crucial to understanding why some precancerous lesions progress to cancer while others do not, why some cancers are highly aggressive while others are indolent, and why some respond robustly to treatment and others do not [7].

One of the key insights into cancer progression to derive from high-throughput sequencing studies is that mechanisms of somatic evolution can differ widely across cancers. Mechanisms of somatic hypermutability may differ between distinct patients for a single cancer type [8] and even between distinct cell lineages [9] or over time [10] in a single tumor. Different cancers may be prone to varying degrees of point mutation hypermutability, microsatellite instability, or chromosome instability [11]. Even within these broad classes, there are now numerous recognized mutational phenotypes presumed to be caused by distinct hypermutability mutations. For example, approximately thirty point mutation signatures [12, 13] are known to exhibit variability in different cancers, with several either known to be connected to specific kinds of hypermutability defects (e.g., pol-*ϵ* defect [14], APOBEC defect [15], or various DNA mismatch repair defects such as those are induced by germline *BRCA1* or *BRCA2* mutations [16]), as well as distinct signatures of copy number or structural variation mechanisms [17, 18], such as those due to *TP53* dysfunction [19, 20]. At present, a number of these hypermutability signatures remain of unknown origin [12]. It remains elusive whether others might be detected as we gain better power to resolve broader classes of mutations and precisely quantify them via deep sequencing [21]. A variety of lines of evidence have suggested that these distinct hypermutability phenotypes have important implications for how a tumor is likely to evolve in the future. For example, it has been shown that tumors prone to copy number alterations (CNAs) and aneuploidy via whole genome duplication (WGD) have significantly worse prognoses than similar tumors only prone to focal CNAs [22–24]. Similar observations have appeared anecdotally for a variety of specific mutation classes.

Here, we sought to explore a vital implication of these past studies: how a tumor is likely to progress in the future is influenced by, and in principle predictable from, the mechanisms by which it has evolved so far, independent of the specific spectrum of driver mutations those mechanisms have so far produced. That is, the patient-specific spectrum of mutational phenotypes acting in a given tumor has predictive power for its future progression. For example, evolutionary statistics based on fluorescence *in situ* hybridization (FISH) data [25] are predictive of whether a tumor will go on to metastasize [26], with more nuanced models of mechanism and variation rate leading to enhanced predictive power [27, 28]. Conceptually, the use of mutational mechanisms as predictors is distinct from, and complementary to, the standard “driver gene” model of prediction—that we predict likelihoods of tumor progression based on its specific pattern of mutations or expression changes in genes of known functional significance in cancer [29–31]—which is the basis of much of the current work in genomic diagnostics for cancer. While conventional approaches to finding genomic predictors focus primarily on markers of the “selection” component of clonal evolution, seeking mutations with putative functional effects on clonal fitness, we here seek to understand the role of the “diversification (drift)” component of clonal evolution by profiling phenotypes that affect the degree and kind of mutations a tumor is prone to generate. We develop this idea of evolutionary predictors of progression by applying “tumor phylogenetics”, i.e., the reconstruction of cell lineage histories in single tumors, to derive quantitative estimates of the evolutionary processes acting on those tumors from their phylogenies. The combination of mechanisms of mutation and the degrees to which they act in a given cancer should then, we propose, have independent predictive power from its specific driver mutations for future progression that we can quantify and harness via machine learning frameworks.

The concept “mutational phenotype” (which is captured by “evolutionary feature”) proposed in this work is related to the concepts of tumor mutational burden (TMB) [32], mutational signature [12], and “mutator phenotype” [2], although distinct from each. Previous research sought to characterize hypermutability in tumors by inferring the TMB from either somatic or germline mutation counts, and found that tumors with higher TMB tend to be more responsive to immunotherapies [33]. Our focus here is on characterizing the ensemble of processes generating mutations and the degree to which they act in a given tumor and not on the resulting mutation burden per se. Mutational signatures capture this idea of an ensemble of processes generating distinguishable mutational patterns at the level of simple somatic mutations. Both TMB and mutational signatures consider the cumulative mutation counts in tumors. The evolutionary features proposed in this work are those that quantify differences in how a tumor generates mutational and clonal diversity independent of the specific mutations it has accumulated. Examples include variation across tumors in rates of mutation-generating processes, such as those captured by mutational signatures, and higher-level quantifications of evolutionary trajectories revealed from clonal lineage trees. More concrete examples of evolutionary features can be found in Table G and H in S1 Text. We also note that we use the term “mutational phenotype” here rather than Loeb’s term “mutator phenotype” [2] to describe the range of phenotypes of mutability we seek to quantify, despite the importance of Loeb’s work in establishing the central idea of a phenotype of hypermutability in cancers, in order to avoid confusion with the more specific understanding of Loeb’s mutator phenotype hypothesis.

The remainder of this paper is devoted to implementing and demonstrating a realization of this idea of progression prediction from somatic hypermutability phenotypes with the goal of examining the degree to which a tumor’s future progression is predicted by its mutational phenotypes independent of specific driver mutations. Below, we describe a general framework, which calls specific somatic variations from either whole exome sequence (WES) data or whole genome sequence (WGS) data, and uses a combination of tumor phylogeny models to extract features for use in predicting progression through regularized Cox regression. We demonstrate that mutational phenotypes are indeed significant predictors of progression outcomes, specifically via prediction of overall survival (OS) and recurrence/disease-free survival (DFS) on data from the Cancer Genome Atlas (TCGA) [34] and the International Cancer Genome Consortium (ICGC) [35], including breast invasive carcinoma (BRCA) [8] and lung carcinoma (LUCA) [36, 37]. In each case, we show that predictions from phylogeny-derived features quantifying mutational phenotypes have significant predictive power for progression outcomes and, further, that these phylogeny-derived evolutionary features provide additional predictive power relative to predictions from clinical or traditional driver-centric genomic features alone. The results demonstrate that variability in mutational phenotypes, and thus evolutionary diversification mechanisms, underlie a substantial portion of the risk of future progression of these cancers.

## Results

We first describe the overall workflow by which we extracted evolutionary and other features, and the methods we used to predict tumor progression and estimate the contribution of each feature class to the progression risk. We then provide an analysis of the genomic and clinical features predictive of tumor progression derived from our analysis pipeline. We then take a closer look into the overall feature landscape and interactions among the different feature types. These components then bring us to the major conclusion of our work, namely, features describing how a tumor is evolving contribute substantially to tumor progression risk, underlying a large fraction of the predictive power of other feature types as well as augmenting it. Finally, we expand on that main conclusion to examine the degree to which the additional information provided by evolutionary features can further improve prognostic prediction beyond what is available from traditional clinical and driver features alone.

### Overall workflow

**Fig 1:**
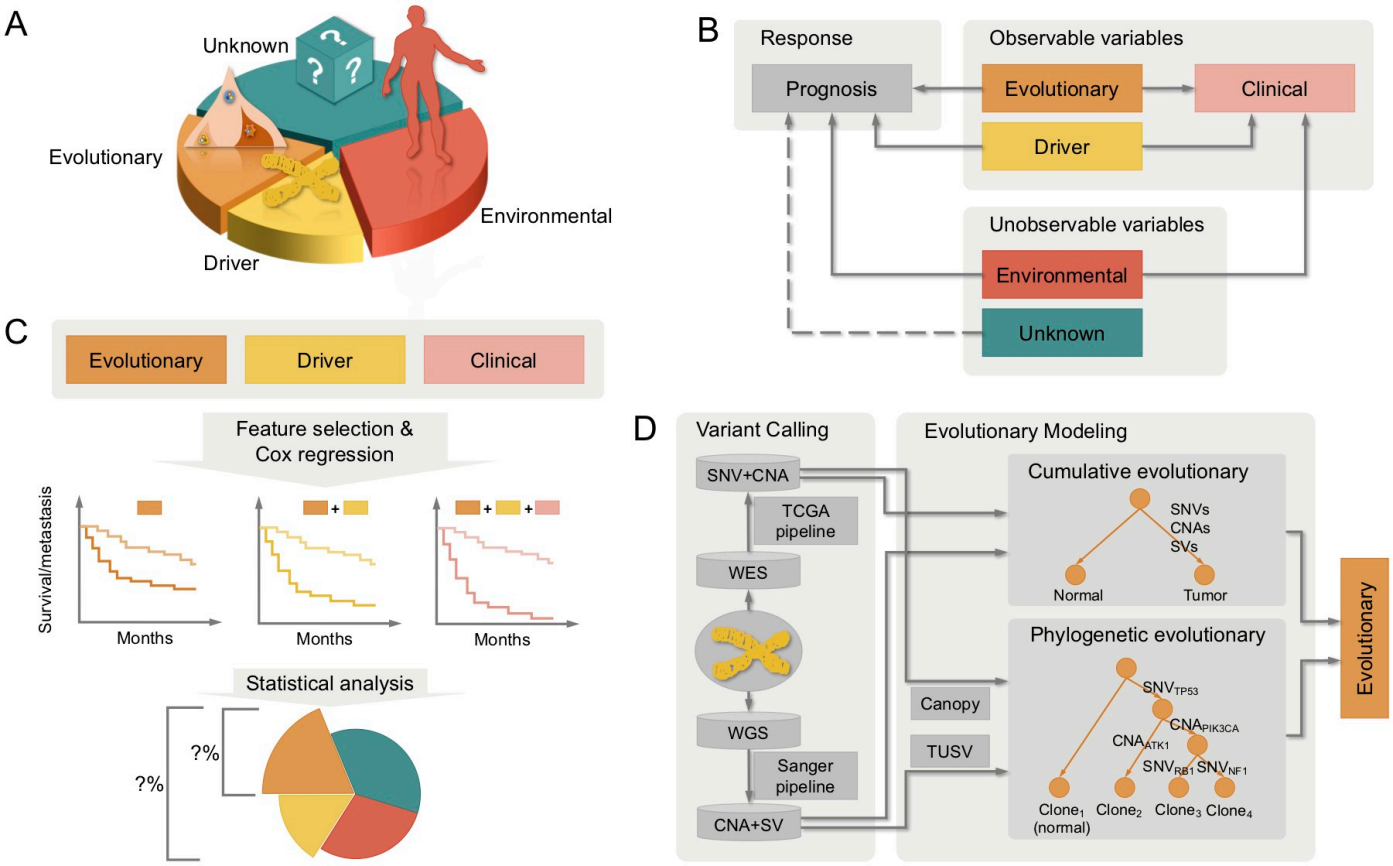
Overall workflow for evaluating the contribution from evolutionary features to tumor progression risk. (**A**) Various factors may account for future prognoses. We categorize these into evolutionary, driver, environmental, and unknown factors. (**B**) Only part of these factors are observable, making the evaluation of contributions complex. In addition, all three known factors have effects on outcomes as well as mutual correlations. (**C**) We conducted Cox regression to predict outcomes of patients, such as survival and recurrence, using combinations of evolutionary, driver, and clinical features, and analyzed their regression results to estimate the contribution of evolutionary features specifically to tumor progression risk. (**D**) The general framework utilizes either WES data followed by standard the TCGA variant calling pipeline or WGS data followed by Sanger variant callers to derive measures of mutational preferences from phylogenetic models of clonal evolution and cumulative mutation burdens.

We assume in the analysis below the future progression of tumors depends on four categories of risk factors: evolutionary, driver, environmental, and unknown factors (Fig 1a). We define evolutionary features as measures quantifying generic preferences for the tumor genome to evolve independent of specific genes affected by these preferences, for example, the overall point mutation rate or the preference for branched versus linear evolution. Driver features capture mutations in specific genes of known cancer relevance, such as point mutations in *TP53* or amplification of *ERBB2*. Environmental features capture additional measurable information about the patient beyond that derivable from the genome, such as demographic features. Unknown factors are presumed risk factors for which we lack measurements.

The general goal of the work is to evaluate the contribution to prognosis predictions from evolutionary features describing mutational phenotypes relative to the independent contributions from other genomic features and to the full set of risk factors. The problem itself is challenging in large part because of the complex correlations between these variables, and the fact that interdependencies between many factors are unknown to us (Fig 1b). For example, we might expect our evolutionary features to include measures of mutational process that are consequences of chromosome instability. These measures will thus be strongly correlated with driver features such as *TP53* mutation that cause chromosome instability. Our goal is to evaluate the portion of such risk attributable specifically to the mutational phenotypes, independent of other consequences of those mechanisms.

We propose, indirectly, to first evaluate the relative contributions of these features to progression risk, through their predictive power in a machine learning analysis using Cox regression, either alone or in combination with additional clinical features and driver-centric genomic features (Fig 1c). We then can use the results of these predictive tests to infer how each kind of feature contributes to overall progression risk. We utilize the *ℓ*_0_-regularized Cox regression model, which is tuned heuristically through step-wise forward feature selection, throughout the work to exploit the power entailed by different sets of features. It achieves better performance than the widely used lasso Cox regression model. We also employ bootstrapping in the Cox model to identify the essential features and capture the interactions across them. The bootstrapping assigns interpretability to the models and is robust to correlated factors.

The process of extracting evolutionary features begins with tumor genome sequencing data (Fig 1d). In principle, these data might be whole exome sequence (WES) or whole genome sequence (WGS), and could be either single-sample (solely tumor data), paired tumor/normal, or multi-sample (multiple distinct tumor sites or regions as well as possibly paired normal). While some study designs might alternatively use targeted deep sequencing data, we would generally consider those data not suited to the present methods, which benefit from profiling larger fractions of the genome to estimate better aggregate mutation rates. We consider here only inference from bulk tumor data [38, 39], although we note that the strategy might be applied to single-cell sequence data [40] or combinations of bulk and single-cell data [41–43], should such data become available for sufficiently large cohorts. The genomic data is preprocessed and passed to one or more variant callers, ideally including single nucleotide variations (SNVs) and copy number alterations (CNAs) calls as well as calls for diverse classes of structural variations (SVs) to produce a variant call format (VCF) file with detected variants and their variant allele frequencies (VAFs) per sample. These variant calls are then fed into tumor evolution algorithms, which deconvolve aggregate bulk data into multiple clonal evolutionary states and infer a cellular lineage tree connecting those states and predicting their likely ancestry. Next, a variety of quantitative measures of the evolutionary process, i.e., “evolutionary features”, corresponding to distinct mutation mechanisms, are extracted. These features intend to approximate the degree to which distinct “mutational phenotypes” are activated in a tumor.

In order to validate the effectiveness and generality of our hypothesis and approach, we compiled two sets of data testing various conditions under which the approach might be applied (Table 1). These datasets cover: two data sources: TCGA [34], ICGC [35, 44]; two cancer types: BRCA [8], LUCA [36, 37]; two sequencing strategies: WES, WGS; two variant callers: TCGA pipeline [34, 45–47], Sanger pipeline [48]; two phylogenetic methods: Canopy [49] (SNV+CNA), TUSV [50] (CNA+SV); two prognostic prediction tasks: OS, DFS. We select breast and lung cancers for validation primarily because they are the most common cancer types and have relatively large TCGA and ICGC cohorts. We treat the two datasets separately and assume all the samples within the same dataset are processed with the same experimental protocol. This avoids the potential confounding factors that may hide behind the datasets. For example, samples from WGS ICGC cohort generally exhibit much greater genome coverage than those from the WES TCGA.

**Table 1:** Statistics of the experiments and datasets in the study.

Dataset	Seq Strategy	Cancer Type	Variant Caller	Phylogenetic Model	Size	#Event/#Censored	
						OS	DFS
TCGA	WES	BRCA	TCGA	Canopy	1044	145/897	102/764
		LUCA	TCGA	Canopy	512	180/323	180/266
ICGC	WGS	BRCA	Sanger	TUSV	90	17/73	14/59
		LUCA	Sanger	TUSV	89	44/43	29/38

We collect two sets of data covering two cancer types (BRCA, LUCA), two experiment strategies (WES, WGS), two variant callers (TCGA pipeline, Sanger pipeline), two phylogenetic models, and two prediction tasks (OS, DFS).

### Informative features for predicting tumor future progression

**Fig 2:**
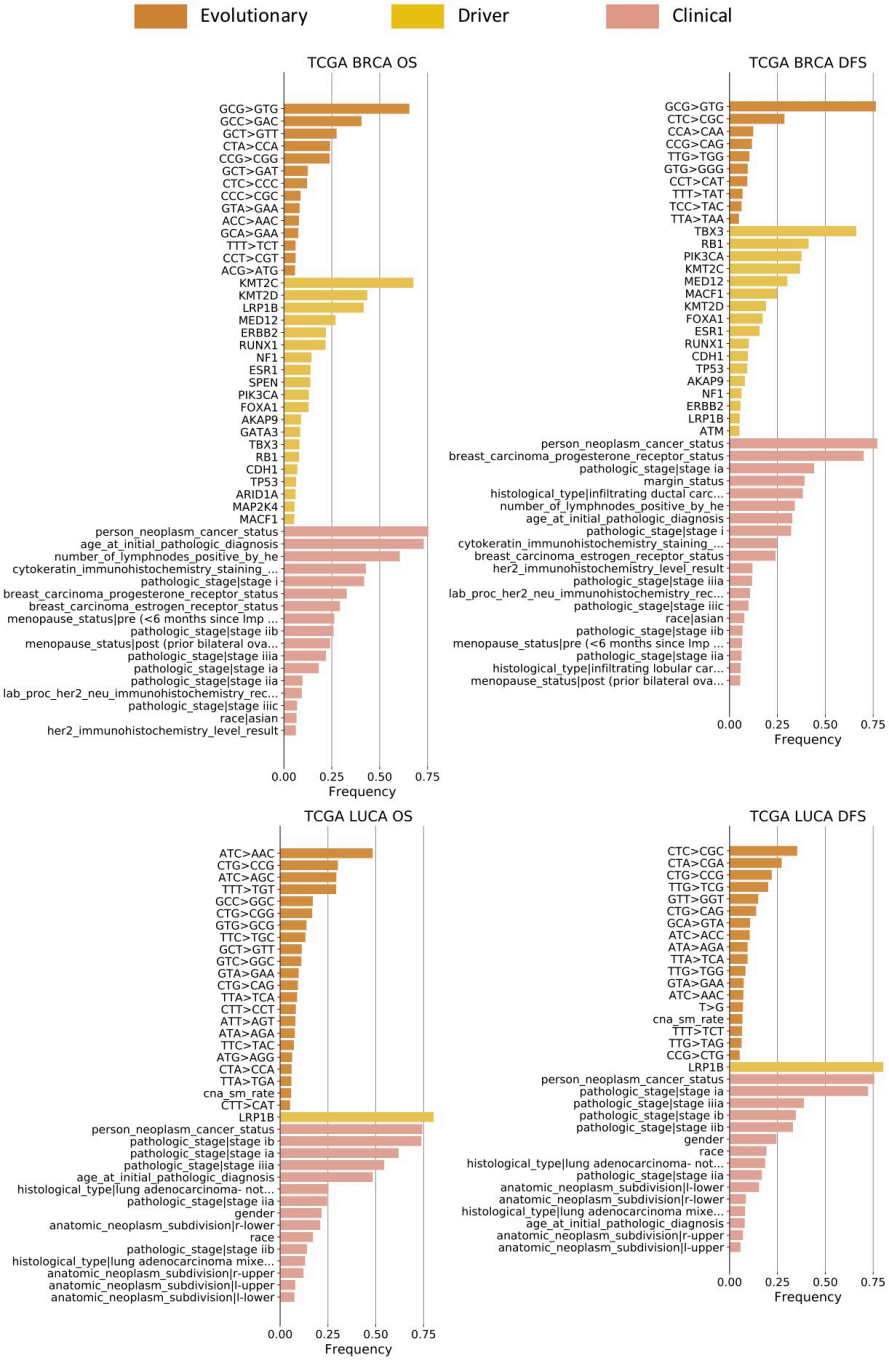
List of important features predictive of prognoses (TCGA dataset). The evolutionary, driver, and clinical features were fed separately as the candidate features for subset selection. We bootstrapped through cross-validation for 1,000 trials, each time with 80% features and 80% samples drawn with replacement. The importance of features is reflected as their frequency of being selected. We show those features among the top ten or selected more than 50 times (frequency>5%). See Fig 3 for important features in ICGC dataset. See Fig A and B in S1 Text for important features when both evolutionary, driver, and clinical features were fed as candidate inputs at the same time instead of individually. See Fig 4 and Fig C in S1 Text for correlations of these important features.

**Fig 3:**
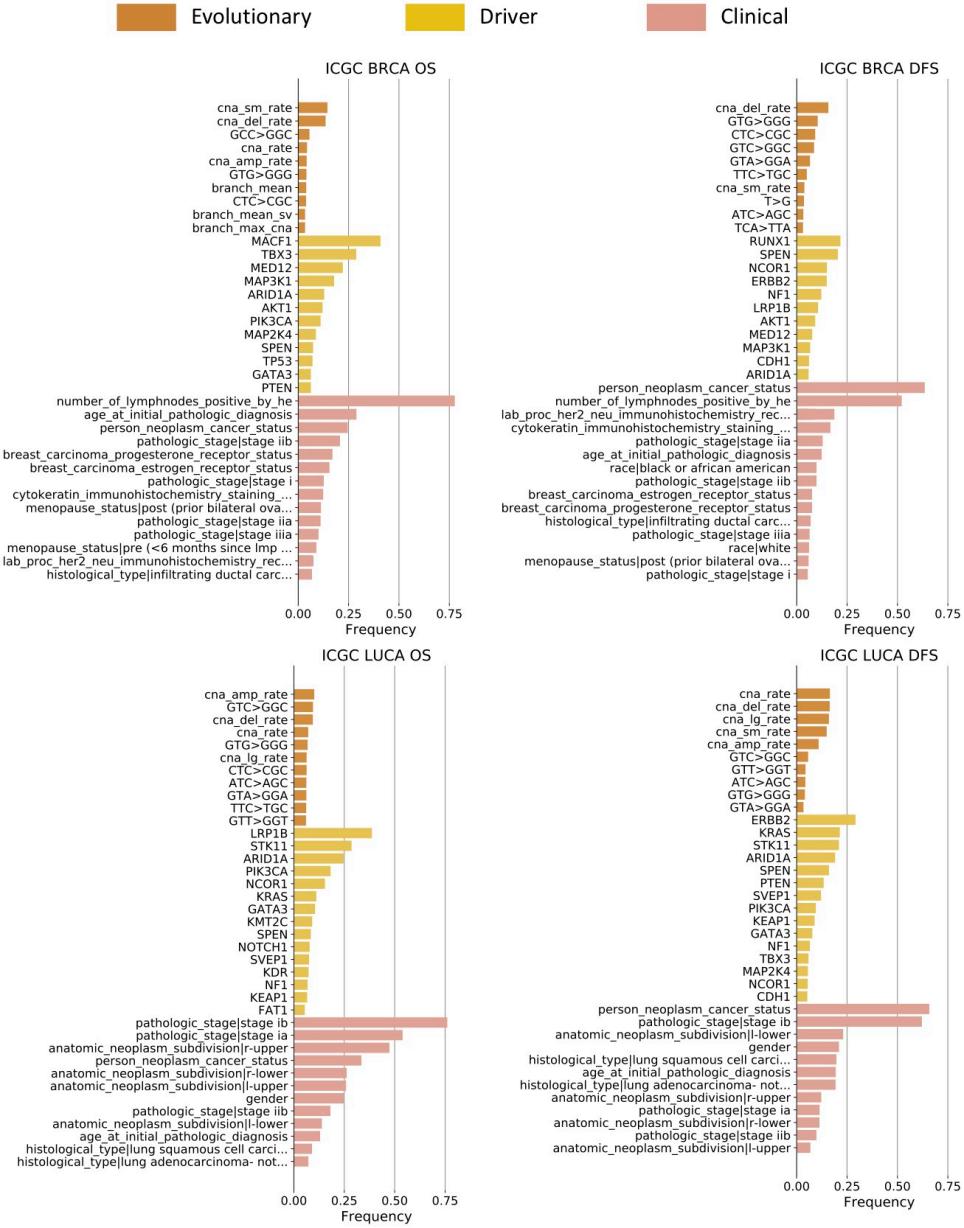
List of important features predictive of prognoses (ICGC dataset). See Fig 2 for important features in TCGA dataset and more detail on how we calculated the selection frequency.

We have around 100 to 200 features in total in each dataset (Table G-J in S1 Text). However, not all these features may be predictive of the future progression of tumors. Therefore, we first set out to evaluate their importance individually. We provided evolutionary, driver, and clinical feature sets separately as candidate features, and fed them into the *ℓ*_0_-regularized Cox regression model for 3-fold cross-validation and feature selection. We conducted bootstrapping by drawing 80% features and 80% samples with replacement for 1,000 trials. These replicates provide the frequency with which each feature is selected. We treat this frequency as a measure of the degree to which a specific feature is informative in the model (Figs 2 and 3), instead of performance or regression coefficient.

There exist alternative ways to evaluate feature importance. A straightforward approach is to conduct univariate regressions for all features and take the concordance index performance as the importance metric. However, a feature that helps achieve the best performance together with other features may not be significantly effective alone in univariate regression. Therefore, it is necessary to use multivariate regression to identify these features. A second solution is to conduct a round of cross-validation using multivariate Cox regression to find the selected subset of features and take their regression coefficients as the effect on the prediction. However, if two features are highly correlated and both are important, the optimal feature subsets are not unique. Moderate correlation can problematic as well, leading to unreliable estimation of the coefficients. The bootstrapping combined with the multivariate regression take advantage of both capturing complex interactions between features and responses (compared with univariate regression) and robustness of selecting features (compared with a single run of the multivariate model). Bootstrapping also has the advantage when potential strong factors exist, such as tumor status, in preventing those strong features from obscuring effects of weaker features. The importance order of most other features will change a little after removing it.

#### Evolutionary features

**Fig 4:**
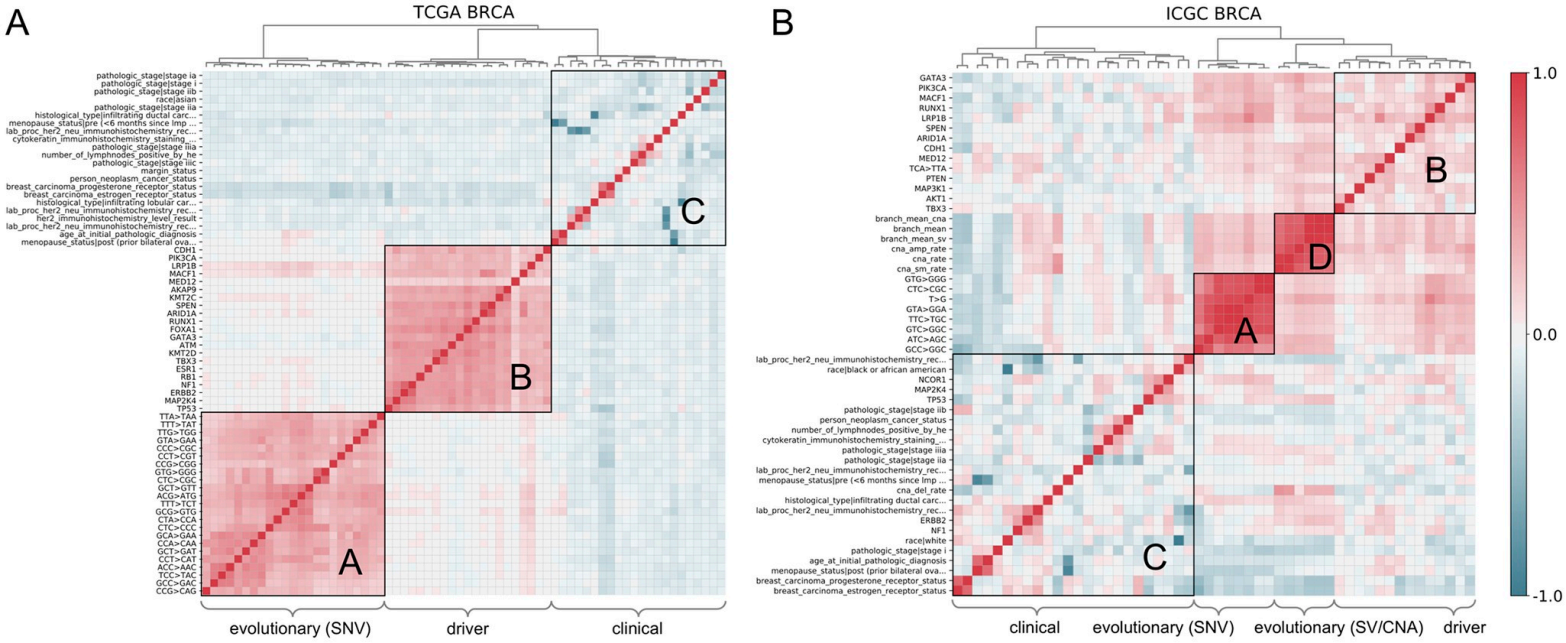
Pearson correlation heatmap for evolutionary, driver, and clinical features of BRCA samples. (**A**) TCGA dataset, (**B**) ICGC dataset. We merge the important features from Fig 2 (TCGA BRCA OS/DFS) and from Fig 3 (ICGC BRCA OS/DFS). Therefore, these shown features can effectively predict either OS or DFS in BRCA samples. We only show the feature name along each row, while the features of columns are in the same order as rows, due to the display limit. (**A**) Strong correlation within each feature type is observed: block-A (driver), block-B (SNV-related evolutionary), and block-C (clinical). Meanwhile, genomic features are more independent and orthogonal to clinical features. (**B**) One can observe an additional block-D (CNA-related evolutionary) in the ICGC data. See Fig C in S1 Text for similar analyses on LUCA samples.

The most strongly predictive evolutionary features vary by dataset, tumor type, and task. Some trinucleotide SNV mutation rates show up as significant in most cases examined (Figs 2 and 3 evolutionary bars) [2]. Trinucleotide SNV rates of the form *N*_*l*_CN_*r*_→*N*_*l*_TN_*r*_ are especially important for the breast cancer prognostic prediction. *C*→*T* (and, equivalently, *G*→*A*) preferences are associated with several mutational signatures [12] and we would hypothesize that their association with age-related signatures largely accounts for their predictive power here (Fig 4a).

There exist systematic variances across the TCGA and ICGC datasets, which reflect the differences in the sequencing techniques (WES vs. WGS), variant caller pipelines (TCGA pipeline vs. Sanger pipeline), and phylogenetic models (Canopy vs. TUSV). SNV-related trinucleotide features seem to be less effective predictors for ICGC than TCGA. It might be caused by the different variant callers used with the two datasets. ICGC utilizes the Sanger pipeline to call simple somatic alterations, which identifies most of these alterations as indels instead of SNVs. Therefore the estimation of the SNV-related evolutionary features is less accurate and informative in the ICGC dataset. One this other hand, the WGS-based ICGC dataset allows for better coverage and profiling, especially with respect to structural variations (SVs) and the copy number alterations (CNAs) that result from them. In the ICGC data, we observe several measures related to SV and CNA rates, which are difficult to capture accurately with WES data but visible in WGS experiments (Fig 3 evolutionary bars), such as average branch length in unit of SV rates (*branch mean sv*), CNA duplication/deletion rates (*cna amp rate*, *cna del rate*), and rates of CNA above/below 500,000 nt (*cna lg rate*, *cna sm rate*). Their appearance indicates that the CNA rates and various sub-categories of it are broadly important predictors of progression. It is consistent with prior knowledge that SVs are a crucial mechanism of tumor progression and functional adaptation through their role in creating CNAs as well as fusion genes [51] and contribute substantially to tumor evolution [50]. In addition, phylogenetic evolutionary features such as the average of branch lengths (*branch mean*) are informative in the ICGC BRCA dataset, suggesting that overall measures of evolutionary heterogeneity inferred by TUSV are important predictors of breast cancer progression and prognoses [26, 52]. Frequencies with which predictive features are sampled are generally lower for the ICGC versus TCGA analyses across feature classes, which likely reflects the much smaller cohort available for the WGS analysis.

#### Other features

Additional analysis of informative driver features, clinical features, and full feature sets is provided in Section A.1 in S1 Text.

### Landscape of evolutionary, driver, and clinical feature spaces

After identifying and analyzing the important features related to future tumor progression, we explored the landscape of evolutionary, driver, and clinical features in their individual and joint feature space. We were especially interested in understanding the relationship between the three types of features and characterizing any internal structure embedded in the tumor evolutionary feature space. We first explored the overall correlation structure of the full feature space to identify the orthogonal features and provide biological insight into subgroups of features. We calculated the Pearson correlation coefficient between each pair of important features (Figs 2 and 3), and performed hierarchical clustering based on Ward distance [53] to group them. We mainly focus here on BRCA samples in both TCGA (WES) and ICGC (WGS) datasets (Fig 4) as they are qualitatively similar to the LUCA datasets (Fig C in S1 Text).

The correlation landscape of BRCA features in TCGA (Fig 4a) and ICGC datasets (Fig 4b) allows one to distinguish three or four closely related blocks of features along the diagonal, corresponding to SNV-related evolutionary features (block-A), SV/CNA-related evolutionary features (block-D), driver features (block-B), and clinical features (block-C), which we analyze in turn. **SNV-related evolutionary features (block-A)**: Most of the SNV-derived evolutionary features are collapsed into a single high-correlation block, essentially corresponding to point mutation rates. This correlation can be easily understood, since all of the features in the block capture overall SNV rate in various ways, although there is some substructure consistent with distinct point mutation processes. These also show some cross-correlation with both driver and SV/CNA features. **SV/CNA-related evolutionary features (block-D)**: This block primarily collects CNA rate features, which we note are highly correlated with one another and partially correlated to both driver and SNV features. This block emerges only in the ICGC datasets, as we would expect since we require WGS data to profile these features accurately. It shows cross-correlation with both SNV and driver features. The correlation landscape is similar to the sub-blocks of block-A and block-D when only evolutionary features are used for generating the heatmap. **Driver features (block-B) and clinical features (block-C)**: Additional analysis of the landscape of driver and clinical features is provided in Section B.1 in S1 Text.

We further sought to visualize any finer higher-dimensional correlation structure of evolutionary features that is not apparent from pairwise correlation analysis (Sec. S3). We utilized tSNE to reduce the evolutionary feature into two dimensions [54] to visualize the feature space as low-dimensional manifolds. We observe a low dimensional manifold structure in the evolutionary feature space that is related to the future progression of tumors (Fig D in S1 Text). For example, there exist distinct islands in the evolutionary feature manifold structure for both BRCA and LUCA samples that are related to poor or optimistic prognoses. This suggests that there are higher-dimensional dependencies in the evolutionary feature space that carry predictive value for progression outcomes.

In general, we may conclude that: 1) The features from each of the four main feature blocks are highly correlated to other features of the same block, validating that it is necessary to prevent collinearity by employing feature pruning and bootstrapping. 2) The genomic features are, in general, slightly negatively correlated to the clinical ones. This relationship is generally stronger in the ICGC data (Fig 4a vs. 4b; Fig C in S1 Text a vs. b). This indicates that the genomic features capture some of the information within the clinical features. 3) Each of the major feature blocks carries independent information despite the correlations between blocks. This independence is especially true for genomic features vs. clinical features. Therefore, one may conclude that the generical genomic features are roughly orthogonal to the clinical features and can be used as complementary in the task of prognostic prediction to improve the performance possibly. 4) SNV and SV/CNA features carry largely orthogonal information to one another despite some cross-correlation. This analysis is concordant with findings of Dawson et al. [55] that suggested a partitioning of breast cancers into distinct classes of SNV-driven and CNA-driven tumors (Fig 4b), although our findings support a subtly different model that these are two orthogonal feature classes both of which may act to different degrees in the same tumors as opposed to defining two orthogonal classes of tumors.

### Evolutionary features contribute substantially to progression risk

In this section, we focus on the central question of this work: to what degree is the future progression risk of the tumor determined by its mutational phenotypes, as captured by evolutionary features. To ask this question, we need to account for the fact that correlations among feature classes will cause non-evolutionary features to serve as at least partial proxies for the evolutionary features we wish to assess. We thus need to assess the contribution of evolutionary features in the context of other genomic features (driver) and other environment-affected features (clinical features). We evaluate the contributions of evolutionary features to the prognostic prediction using a fraction metric inferred from the hazard ratio (HR) between predicted benign and malignant cohorts of tumors on the test set (Fig 1c). We focus on the log-HR-based fraction metric in this work, since it is linear in the scale of feature space. Although it is possible to conduct the similar analysis based on the results from concordance index (CI; Table 2), the nonlinearity of the CI makes the results hard to compare and interpret.

We conducted two-loop cross-validation experiments using evolutionary features, genomic features, or full features, replicating each experiment five times. The HRs (Table A in S1 Text) were then calculated on the test sets that used the three feature sets following Eq (8). We finally estimated the log-HR-based fractions of either evolutionary or genomic features from the log-HRs following Eqs (7) and (9).

**Fig 5:**
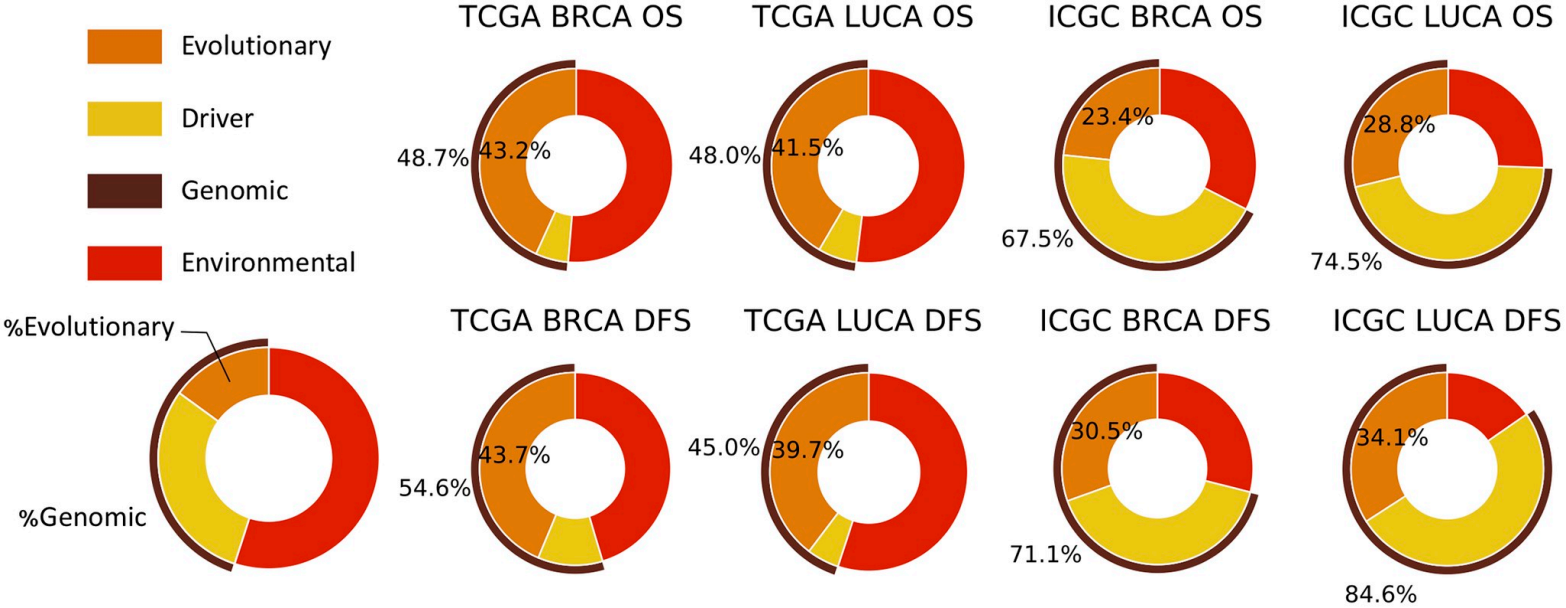
Contribution percentage of evolutionary and genomic features to tumor progression risk prediction. Evolutionary features contribute to around 40% in TCGA data, and 25-35% in ICGC data. The genomic features contribute to around half in TCGA, and 70-80% in ICGC dataset. We estimate the fractions using Eqs (7) and (9) and data from Table A in S1 Text. See Fig F in S1 Text for fractions where neoplasm status is removed from clinical features.

Fig 5 shows that the evolutionary features contribute around 40% of the risk captured by the predictive models for both the BRCA and LUCA in the TCGA dataset and around 25%-35% of the risk for BRCA and LUCA in the ICGC dataset. We believe the difference largely occurs, despite the better genome coverage in the WGS-based ICGC data, because the Sanger variant calling pipeline identifies most of the simple somatic mutations as indels instead of SNVs, preventing us from effectively extracting the trinucleotide features that proved to be among the most powerful evolutionary features for tumor progression prediction (Figs 2 and 3). While the exact numbers are thus contingent on the data available and how it is processed, the data suggest that mutational phenotypes, corresponding to variability in mechanisms of evolutionary drift, account for approximately a third of the total progression risk identified.

Genomic features in total contribute around 50% of the risk in the TCGA dataset, and around 70%-80% in the ICGC data. The discrepancy is mainly because driver features proved substantially more predictive for ICGC than TCGA. It appears to occur because of the sparsity of the driver features in the TCGA WES dataset. This is especially true for LUCA, where in most cases only a single driver *LRP1B* has more than 5% non-zero values (Fig 2 TCGA LUCA driver bars). In addition, the driver features in TCGA are more highly intra-correlated than those in ICGC (Fig 4a vs. 4b). ICGC WGS has a higher coverage than TCGA WES, and since all the somatic alterations that happened within the genome region of drivers, including SNVs, indels, CNAs, and SVs are counted as driver mutation, ICGC has a substantially richer representation of the driver features. While the exact numbers are again dependent on the specific data, the results suggest that very roughly half of the contribution of genomic features to progression risk comes from variability in mutational phenotypes and half from other independent genomic factors.

We note here that we do not provide the contribution fractions of drivers by themselves, although these can be similarly calculated from Table A in S1 Text. The summation of evolutionary fractions and driver fractions does not necessarily equal the genomic fractions, since we have to take into account the correlation and interaction between the two feature sets. That is, some portion of the driver risk is explained by driver mutations that induce hypermutability phenotypes, and our approach is intended to separate that information from driver risk that is not caused by mutational phenotypes.

Both breast and lung cancer patients have distinct survival and recurrence probabilities for different neoplasm status (*person neoplasm cancer status*; Fig E in S1 Text) and it is the single strongest predictor of outcome, but its availability could be considered unreasonable to assume in normal practice. We were therefore interested in how the models behave without that one feature. When we exclude the tumor status clinical feature (denoted with “Δ”), the contribution fractions of evolutionary and genomic features both increase further (Table B and Fig F in S1 Text) by up to approximately 20%.

### Evolutionary and genomic features improve prognostic prediction

While improving predictive power for progression is not the central goal of this study, the guiding hypothesis nonetheless suggests that evolutionary features should provide predictive power for future progression, an inference that we test in this section. Although it is to be expected that clinical features will have the strongest individual predictive value among feature classes, we are interested here in establishing whether evolutionary features provide any additional predictive value for progression outcomes beyond that provided by clinical features alone or clinical features supplemented by driver genomic features. We use the two groups of evaluation datasets (TCGA WES data with more samples; ICGC WGS data but with smaller cohort size) to explore different settings and possibilities in clinical practice (Table 1), such as different cancer types, sequencing methods, and prediction tasks, to answer that question. The results indicate that evolutionary features can incrementally enhance the predictive power in most cases (Table 2) and that genomic features (evolutionary + driver) collectively enhance predictive power relative to clinical alone.

**Table 2:** Performance of prognostic prediction with different features in WES-based TCGA and WGS-based ICGC samples.

	TCGA (WES)				ICGC (WGS)			
	BRCA		LUCA		BRCA		LUCA	
CI	OS	DFS	OS	DFS	OS	DFS	OS	DFS
evolutionary	56.9±0.56	53.3±0.44	51.8±0.26	50.5±0.25	51.7±0.74	54.0±1.21	52.9±0.73	53.2±0.66
driver	54.9±0.54	55.4±0.59	53.6±0.05	53.6±0.03	53.1±0.97	51.1±0.46	51.2±0.59	50.4±0.59
genomic	59.2±0.49	56.2±1.01	53.2±0.35	51.7±0.38	57.5±2.15	56.2±2.30	52.8±1.28	54.5±1.03
clinical	78.9±0.29	74.3±0.51	67.4±0.31	63.1±0.35	75.0±1.47	70.3±2.47	**62.1**±1.14^ns^	58.1±1.54
full	**79.7**±0.32**	**75.1**±0.69*	**67.9**±0.47^⋅^	**64.4**±0.35***	**80.7**±1.34***	**80.8**±3.22**	61.7±1.09	**61.5**±2.65*

Clinical feature set performs best among the evolutionary, driver, and clinical feature sets. However, the additional genomic features are synergistic and promote the prediction of prognoses (“full” features), except the ICGC LUCA OS task, where two feature sets are on par with each other. We evaluate the performance using two-loop cross-validation (CV). Both TCGA and ICGC utilize 3-fold outer CV to calculate the concordance index (CI) for evaluation. We repeat the outer CV for five times to calculate the means and standard deviations to measure the variation on each test. As for inner CV, we use 3-fold CV for TCGA, and leave-one-out CV (LOOCV) for ICGC since it has a smaller sample size. “Genomic” means all genomic features, including driver and evolutionary features. “Full” means both evolutionary, driver, and clinical features. Statistical significance notation for the “full” vs. “clinical” is defined by the one-sided test *p*-value.

^ns^: not significant;

^⋅^: *p* < 0.10;

*: *p* < 0.05;

**: *p* < 0.01;

***: *p* < 0.001.

See Table D in S1 Text for results where neoplasm status is removed from the clinical and full feature sets. We refer interested readers to Fig A and B in S1 Text for features selected in the “full” experiments (last row of the table).

We utilized two-loop cross-validation (CV) to tune, train and unbiasedly evaluate the performance of *ℓ*_0_-regularized Cox regression on different sets of features with the performance metric of concordance index (CI). We used 3-fold CV in both TCGA and ICGC for outer loop CV; 3-fold CV in TCGA, and leave-one-out CV (LOOCV) in ICGC for inner loop CV, since ICGC has far fewer samples. In order to quantify the uncertainty resulting from random choices in splitting the datasets and assess the robustness of the methods, we randomly shuffled the samples and repeated the experiments for five times to derive the means and standard deviations of prediction outcomes.

As expected, the clinical predictors provide the strongest predictive information among any single predictive class (evolutionary, driver, and clinical), as assessed by CI for both WES-based TCGA and WGS-based ICGC data (Table 2). Evolutionary and driver features alone are predictive as well but substantially less than clinical features individually. Evolutionary and driver features perform comparably to one another, with each performing slightly better than the other in half of the scenarios. In most cases, genomic features collectively performed better than either evolutionary or driver alone, suggesting that each carries some orthogonal information. In all but one case, the full feature set outperformed clinical features alone. Among all the eight cases, six show statistically significant improvements (*p*-value<0.05), and two show effective ties in performance between clinical and full features (TCGA LUCA OS, ICGC LUCA OS) suggesting that the additional genomic features are already largely explained by the information captured by the clinical features. Collectively, the results suggest that each of the three feature classes carries at least some predictive power not captured by the other classes.

The selected features from the full feature sets show that the clinical features are always the most essential in the model (Fig A and B in S1 Text). However, the SNV-related evolutionary features mainly facilitate the model for the TCGA dataset while driver features and CNA-related features mainly facilitate the model in ICGC dataset. Similar to the above section, we hypothesize that this reflects the advantages of WGS in capturing the somatic alterations in the driver regions. However, the Sanger pipeline takes most of the simple somatic mutations as indels instead of SNVs, leading to a loss of information when we infer the SNV-related evolutionary features. This variability between the different variant calling pipelines used by the two sequencing projects suggests the importance of feature engineering for the prognostic prediction task.

As mentioned previously, the tumor status clinical feature is highly related to cancer prognoses. We therefore conducted parallel experiments in the absence of that feature (dubbed “clinicalΔ”, or “fullΔ”≔clinicalΔ+genomic). We find a qualitatively similar result that prediction from clinical features is still superior to that driver or evolutionary features alone, although to a lesser degree. In all but one case, the fullΔ feature combination still outperforms clinicalΔ alone (Table D in S1 Text).

**Fig 6:**
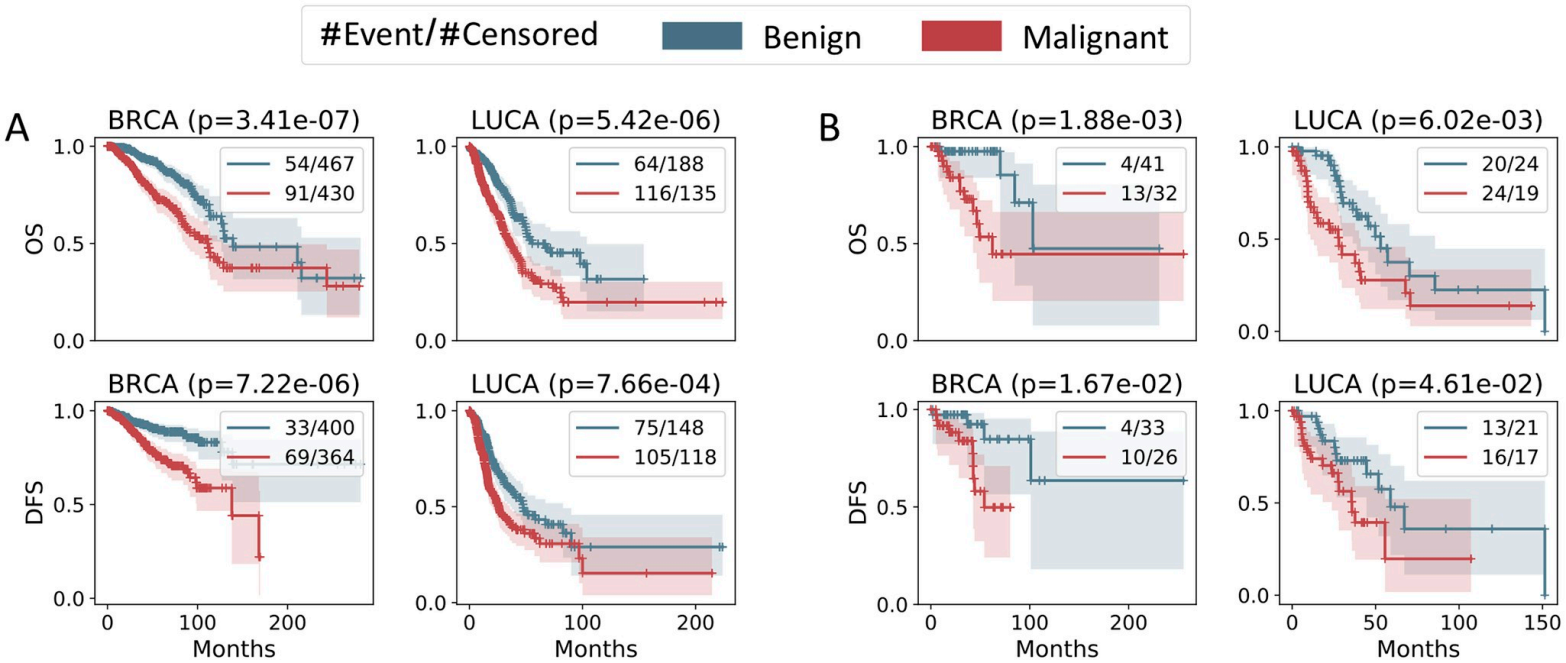
Kaplan-Meier estimators of predicted malignant and benign cohorts using both clinical and genomic features. (**A**) TCGA, (**B**) ICGC. Logrank test shows significant distinct survival and recurrence profiles between the two cohorts across both datasets and cancer types.

Finally, we evaluated the ability of the best combinations of full features in both TCGA and ICGC to stratify patients with distinct survival/non-recurrence time as assessed by the logrank test (Fig 6). Patients were split into two groups: malignant and benign, based on the predicted hazards of the events (decease or recurrence) using *ℓ*_0_-regularized Cox model at the outer CV phase. Samples with predicted hazards larger than the median of predictions were classified as malignant, otherwise as benign. Fig 6a illustrates the separations by OS and DFS time between the predicted malignant and predicted benign tumors in the WES-based TCGA through distinct Kaplan-Meier estimator curves. The separations are statistically significant for both breast and lung cancers. Similar significant separations exist for tumors in the WGS-based ICGC dataset (Fig 6b).

We were also curious whether other Cox regression variants might perform better than *ℓ*_0_-regularized Cox model on our datasets, e.g., lasso. We therefore performed the experiments following the same two-loop CV protocol using lasso for feature selection as well. We tuned the *ℓ*_1_ coefficient of lasso (λ ∈ [0.03, 30.0]) in the inner CV. One can see that our *ℓ*_0_-regularized Cox regression model performs better than the lasso in most cases (Table 2 vs. Table E in S1 Text). In addition, we noticed that it is difficult for the lasso model to fully utilize the additional genomic features when integrating them with the clinical features (Table E in S1 Text row “full” vs. “clinical”). From our observations on the lasso regression results, we find they are more sparse than the *ℓ*_0_-regularized method, indicating that lasso might be too strict in its feature selection to make full use of the small gains from individual genomic features. When both genomic and clinical features are provided, lasso tends only to keep the clinical features.

In summary, these experiments indicate that genomic and clinical features act synergistically to improve the prediction of prognoses, and that in general, adding evolutionary features that capture variation in mutational phenotypes of tumors enhances predictive power relative to clinical features and driver features. Note that this analysis asks a different question than the hazard ratio analysis in the previous section. In the preceding section, our goal was to estimate the portion of risk entailed by mutational phenotypes specifically, which is captured by evolutionary features but may also be redundantly captured by the other feature classes. The analysis in this section explores the non-redundant information captured by each feature class beyond what is carried by the others. Collectively, these two results suggest that a large fraction of progression risk is captured by evolutionary features, and while much of that information about mutational phenotypes is also captured by driver and/or clinical features, there is additional information in the evolutionary features that manifests as somewhat enhanced predictive power.

## Discussion

Our work supports the central hypothesis that the variation in mutational phenotypes accounts for a large fraction of cancer progression risk, under various confounding environmental factors, beyond the risk for which independent of clinical factors and specific driver gene mutations account. The recognition that cancer is a product of somatic evolution has proven greatly influential in our understanding of how cancers appear and progress. However, practical application of evolutionary theory in cancer has predominantly focused on the “selection” side of evolution, e.g., identifying functional mutations under selection as biomarkers or drug targets, and less so on variations cancer-to-cancer in mechanisms of “diversification”, or the drift component of evolution. The exact quantification of various sources of risks presented here must necessarily be considered preliminarily, as it depends on the cohorts examined, the data sources available, the kinds of mutability mechanisms and other features profiled, and the details of the machine learning analysis. Nonetheless, the results suggest that the processes by which a particular tumor generates non-specific genetic diversity (the drift component of tumor evolution) are of comparable importance to the specific functional mutations that diversity has produced (the selection component of tumor evolution) in determining whether or not the tumor progresses. This observation has implications for how we pursue diagnostics for cancers or precancerous conditions, prognostic prediction, and treatment strategies.

Nonetheless, large gaps remain in exploring in detail the space of predictors, the mechanisms by which they act, and the best strategies to realize their translational potential. Some of these questions remain data-limited and likely cannot be answered without larger cohorts and richer clinical metadata. Our work suggests that WGS provides an advantage over WES in improving prediction power beyond that of clinical and driver-centric features, particularly in allowing us to better capture the important role of SV/CNA-driven mutability in parallel to SNV-driven mutability. Available cohorts of patients with WGS data are still limited, an issue that proved a limitation to the present work despite our use of some of the largest cancer WGS corpora that have been made accessible to researchers. Insufficient sample sizes could lead to fragile models and statistically insignificant performance.

Another area where the work can likely be advanced is with respect to describing patient-specific mutational phenotypes effectively through a set of partially independent variables. Our results suggest that SV/CNA and SNV mutator phenotypes act largely independently of one another (block-A and block-D in Fig 4, Fig C in S1 Text). Prior literature suggests a finer structure underlying these two broad types of processes exists. In the SNV domain, mutational signature analysis has demonstrated the existence of approximately 30 independent point mutation processes acting to different degrees on different cancers [12]. We likewise know that distinct mechanisms of SVs and CNAs act on a tumor genome [56] and at least some of these, such as whole genome duplication (WGD), are independently predictive of outcome [22–24]. Our analysis of the evolutionary feature space suggests that there is a structure of the manifolds describing mutability phenotypes related to progression outcomes, but more work is needed to develop more interpretable models of this manifold structure and translate that into both better predictions and mechanistic insights.

There are many avenues by which the interpretability and predictive power of the model might be promoted. First, although our analysis demonstrates through permutation tests that predictive power of the current evolutionary features is not just noise or a data artifact (Fig G and Table F in S1 Text) and shows that the current evolutionary features provide improvements in predictive power when added to clinical and driver features, we nonetheless observe systematic variances of evolutionary and driver features in the same cancer type across the two datasets (Figs 2 and 3). Such variances mainly came from different sequencing coverages, variant callers, and phylogenetic models at the time of data extraction and collection. Our conclusions on the selected informative features are thus mainly restricted to the data extracted with the same platforms. We might further generalize the informative features, reduce variations of models, and improve the overall performance through ensemble learning [57], by integrating the results from different sequencing techniques, variant calling pipelines, and clonal inference algorithms. Second, through unbiased evaluations of the two most common cancer types under various settings, we found qualitatively similar results on the contributions of evolutionary features. However, pan-cancer analysis across different cancer types given sufficiently large cohorts can be a future direction to consolidate and extend this observation, and enable us to evaluate the different tumor evolution mechanisms acting in each cancer type. Third, more advanced methods for survival analysis might be warranted. For example, deep learning has been applied to survival analysis recently [58–60], demonstrating the feasibility of using a neural network as an effective feature extractor for predicting prognoses if the evolutionary, driver and clinical features of all the available samples are integrated properly. In summary, we believe that future work with better feature extraction protocols, improved phylogeny reconstruction algorithms, more extensive and diverse datasets, and more powerful machine learning models will provide us with greater understanding and a more accurate estimation of the risk contribution from the mutational phenotypes.

## Conclusion

Understanding the factors that underlie the risk of cancer progression is a difficult problem with implications for many critical clinical decisions, such as whether to pursue aggressive treatment, which treatment options to consider, and how frequently to monitor patients for signs of resistance or recurrence. In the present work, we conducted a comprehensive study to evaluate the contribution to progression risk, specifically from evolutionary features quantifying the degree to which different mutational processes act on a tumor’s genome. The work estimated that under the different environmental conditions, evolutionary features account for around one-third of the future progression risk of the tumor, and that this information is complementary to and only partially redundant with that derivable from common driver genes and standard clinical metadata. The work thus implies that variability in evolutionary diversification (drift) is of comparable importance to variability in forces of evolutionary selection in determining which tumors progress. We also explored the interdependencies among these features and feature classes, showing a complex correlation structure indicative of the heterogeneity in mutational processes across cancer types and individual cancers and suggesting how this heterogeneity helps to shape accumulations of driver mutations and ultimately clinical presentations of cancers. We further proposed a strategy for enhancing the power for making such predictions through the use of evolutionary features, in addition to clinical data and traditional driver features. We demonstrated via a novel machine learning approach that that generic mutability features lead in most cases to a statistically significant enhancement of predictive power for future progression beyond that offered by clinical predictors and more conventional “driver-centric” genomic predictors.

## Materials and methods

### Variant calling and evolutionary modeling

For each cancer sample, somatic genomic variants were first called by a range of possible tools as discussed below, then the SNVs, CNAs, and SVs were integrated and converted into a single VCF file, which is the input format required by both evolutionary tree methods considered, Canopy [49] and TUSV [50]. Both methods output fractions of clones in each tumor sample, the inferred phylogenetic tree connecting clones, and the acquired somatic variants of clones during evolution along the tree edges.

We made use of calls from two different variant callers in the experiments, according to the sequencing strategy of the samples. For the WES BRCA and LUAD samples from TCGA corpus (Table 1 TCGA), we downloaded SNVs and CNAs from the TCGA Genomic Data Commons Data Portal (GDC) [61]. It provides calls from the TCGA pipeline [34], which uses a consensus of standard variant callers such as MuSE [45], MuTect2 [46] and GISTIC2 [47]. We made use of WGS samples from the ICGC/PCAWG project [35], which provides one of the largest public corpora of WGS samples for breast cancer and lung cancer (Table 1 ICGC). For these samples, we downloaded SNV, CNA, and SV calls [62], which had been computed for this project using the Sanger pipeline [48]. The users can also use other variant callers such as Weaver [63, 64] and novoBreak [65] for the WGS samples. We pooled the two cohorts of lung cancer: LUAD and LUSC into a single LUCA category to increase the size of the dataset.

We applied two general approaches to derive tumor phylogenetic trees, based on the availability of WGS and WES data. We consider the two data types primarily to assess whether the major conclusions of the analysis are robust to the differences in data type, and particularly whether they are manifest in more widely available WES data despite its more limited utility for assessing mutational phenotypes. WGS data provides a much better ability to call SVs. To assess the predictive value of SVs, we sought to capitalize on this capability by building phylogenies using a customized version of the TUSV phylogeny software [50], which infers phylogenies from SVs and CNAs and is, to our knowledge, the only tumor phylogeny program currently able to incorporate SVs into its trees. For WES data, we instead used the third-party tool Canopy [49], which makes inferences from SNVs and CNAs. Interested readers can find more detail on our use of Canopy and TUSV in Section H in S1 Text.

Note that both the clonal phylogenetic models, including Canopy and TUSV, infer the most likely trees based on the sequencing data available. Since the samples available for the patient are limited, the estimation of phylogenies may be noisy and inaccurate. However, We can still take advantage of the useful information hidden behind these phylogenetic evolutionary features of large scale samples through the well-designed machine learning model proposed in the work.

We finally added a trivial evolutionary model, which we dub the “cumulative evolutionary” model, in contrast to the more complex tree models inferred by Canopy or TUSV. This cumulative model is intended as an aggregate approximate model of evolutionary preferences derived from overall mutation burdens. For this model, we assume that there is a single branch of evolution from normal to cancer, resulting in a diploid tree of a “normal” root and single derived cancer state. This two-node cumulative evolutionary tree gives a crude approximation to evolutionary rates that requires minimal assumptions about the underlying evolutionary model and data types.

### Evolutionary feature extraction

Our experiments made use of two types of genomic features (evolutionary and driver features) and clinical features, assumed to be data that would typically be available for diagnosis in clinical practice. We provide details of driver and clinical feature extraction as well as feature preprocessing in Section I in S1 Text.

#### Evolutionary features

We first derived a set of cumulative evolutionary features of samples from overall mutation burdens subdivided by mutation class, and analyzed them as if they were derived from two-node evolutionary trees (Table G in S1 Text). Although we might have directly selected the known mutational signatures as part of the evolutionary features (e.g., *G*→*C* using the COSMIC database), we instead chose to include as many potential evolutionary features as possible including trinucleotide frequencies not corresponding to any common signature. In part, this enabled us to identify the useful features outside of our current knowledge from literature. It also allowed us to consider that informative evolutionary features might be different across different platforms due to different sequencing methods and variant calling pipelines. In addition to the total SNV mutation rates (*snv rate*), we used mutation rates for different types of SNVs, e.g., *T*→*A*. We took *G*→*T*, *G*→*C*, *G*→*A*, *A*→*T*, *A*→*G*, *A*→*C* equivalent to *C*→*A*, *C*→*G*, *C*→*T*, *T*→*A*, *T*→*C*, *T*→*G* since they are equivalent. For example, when a mutation *G*→*T* is observed in one DNA strand, there must be another mutation *C*→*A* in the complementary strand, and we treat the two as equivalent. There are therefore 4 × (4 − 1)/2 = 6 such mutation patterns in total. We also broke these down further into the trinucleotide context, as is typically done in mutational signature analyses [12]. A trinucleotide mutation has the general form of *N*_*l*_N_*x*_N_*r*_→*N*_*l*_N_*y*_N_*r*_, which represents the muation of *N*_*x*_→*N*_*y*_ with the left and right contextual nucleotide *N*_*l*_ and *N*_*r*_. There are 4 × 6 × 4 = 96 trinucleotide mutation features in total. Apart from the mutation rates related to simple somatic mutations, we also estimated various aggregate mutation rates of CNAs and SVs, e.g., *cna rate*, *sv rate*. Similar to the mutational signatures of SNVs, we tried to characterize the copy number-level equivalents by taking into account the size of CNA region and duplication vs. deletion of the CNA, e.g., rates of CNA above 500,000 nt (*cna lg rate*), CNA deletion rates (*cna del rate*) etc. In each case, we treated mutations as occurring on a single evolutionary tree edge spanning from normal to tumor. When the normal state is unknown, we screened out sites of common germline single nucleotide polymorphisms (SNPs) using germline SNP data from the 1000 Genomes Project [66], and used deviation from a standard human reference (GRCh38/hg38 for TCGA, GRCh37/hg19 for ICGC) [67]. We assumed a fixed edge length of ten years as an estimated time from the appearance of the first ancestral tumor cell to the time of sequencing in order to provide a scaling factor to convert mutation counts into estimated rates, although we note that the scale is arbitrary and does not affect the machine learning inference.

Second, we added a set of features derived from the more involved evolutionary trees built by Canopy or TUSV. After a phylogenetic evolutionary tree was built for WES by Canopy, or for WGS by the extended TUSV, measures that quantify topological features of the tree were extracted (Table H in S1 Text). Since the outputs of TUSV include extra SV information, we have some additional phylogenetic features for WGS samples. However, there are still some common tree features, such as the clone number (*num clone*), the height of the phylogeny (*height*) and the average of edge lengths (*branch mean*) that are conserved between phylogeny inference methods.

### Cox regression and its regularized variants

We utilized *ℓ*_0_-regularized Cox regression model throughout this work, using step-wise forward feature selection as a heuristic strategy to tune hyperparameters and select features.

#### Proportional hazards and Cox regression

The clinical prognostic outcomes of cancer patients, such as OS and DFS, are censored data, meaning that the time to a death event or recurrence event was not observed for some samples due to the limited follow-up time. Therefore, instead of conventional classification or regression methods, we performed Cox regression [68] and evaluated the prediction performance with metrics for survival analysis, which are specifically designed to cope with these censored data. In our formulation, the samples are

$$\begin{array}{c} {\left\{\left(X_{i},y_{i},\delta_{i}\right)\right\}}_{i=1}^{N}, \end{array}$$ (1)

where *N* is the total number of samples, $X_{i}\in R^{m}$ is the feature vector of sample *i*, *δ*_*i*_ ∈ {0, 1} indicates the status of patient *i* at the last follow-up time *y*_*i*_ = min(*T*_*i*_, *C*_*i*_): If *δ*_*i*_ = 1, the event happened and was observed at time *y*_*i*_ = *T*_*i*_. If *δ*_*i*_ = 0, the event had not happened at the censoring time *y*_*i*_ = *C*_*i*_.

Cox regression is a semi-parametric regression method based on the proportional hazards (PH) assumption:

$$\begin{array}{c} h_{i}\left(t\right)=h\left(t\right)\cdot \exp(-\beta^{⊺}X_{i}), \end{array}$$ (2)

where *h*_*i*_(*t*) ≔ lim_Δ*t* → 0_Pr(*t* < *T*_*i*_ ≤ *t* + Δ*t*|*T*_*i*_ > *t*)/Δ*t* is the hazard of patient *i* at time *t*, or in another word, the probability of death if the patient has survived to time point *t* (for OS; similarly it is the hazard of recurrence for DFS), *h*(*t*) is the non-parametric part calculated from the training data, *X*_*i*_ is the feature vector of sample *i*, $\beta \in R^{m}$ is the model parameter to be estimated. Instead of predicting whether the patient will be dead or alive, Cox regression estimates *β* and provides the hazard of the patient following Eq (2). When the total number of samples is large, we can roughly assume *h*(*t*) to be close enough at the time of cross-validation (CV). The comparison of *h*_*i*_(*t*) thus reduces to the comparison of risk score *η*_*i*_ = −*β*^⊺^ *X*_*i*_, i.e., the logarithm of hazard’s parametric part exp(−*β*^⊺^ *X*_*i*_), which is independent of time *t* [69]. There exist other approaches as well, e.g., by comparing the expectation of the survival time. However, we found that the comparison of risk score is more robust and reliable in our experiments. We implemented the Cox regression and its variants using the Python package lifelines [70].

#### Cox regression without penalty terms

At the time of training, we optimized the negative log-partial likelihood function of the Cox model:

$$\begin{array}{c} \min_{\beta }\;l\left(\beta \;|\;{\left\{(X_{i},y_{i},\delta_{i})\right\}}_{i=1}^{N}\right). \end{array}$$ (3)

Since the dimension of the features is large and the features are correlated in our application, the lifelines package is unstable in optimizing the objective function Eq (3). In addition, such large feature dimension can lead to severe overfitting, meaning that we can achieve perfect performance on the training set, but the performance is not generalizable to the test set. We considered two Cox variants in this work.

#### ℓ_1_-regularized Cox regression (lasso)

Lasso is one of the most widely used sparsity-promoting regularization methods to cope with the high-dimensional feature space. It adds an *ℓ*_1_ regularization term to Eq (3):

$$\begin{array}{c} \min_{\beta }\;l(\beta \;|\;{\left\{(X_{i},y_{i},\delta_{i})\right\}}_{i=1}^{N})+\lambda {\left\|\beta \right\|}_{1}, \end{array}$$ (4)

where the coefficient λ ∈ {0.03, 0.1, 0.3, 1.0, 3, 10, 30} was tuned through inner CV. We showed that lasso generally performed worse than *ℓ*_0_-regularized Cox regression (Table E in S1 Text).

#### ℓ_0_-regularized Cox regression

Instead of the *ℓ*_1_ penalty, *ℓ*_0_-regularized Cox regression selects the subset of features by optimizing the following objective:

$$\begin{array}{c} \min_{\beta }\;l(\beta \;|\;{\left\{(X_{i},y_{i},\delta_{i})\right\}}_{i=1}^{N}),\;\text{s.t}.\;{\left\|\beta \right\|}_{0}\leq k, \end{array}$$ (5)

where ‖*β*‖_0_ is the *ℓ*_0_-norm that counts the number of nonzero elements in coefficient vector *β*. The integer parameter *k* is tuned at the training and validation phase to achieve the highest inner CV performance. As we will discuss in the Section “Tuning *ℓ*_0_-regularized Cox model: subset selection”, it is computationally infeasible to find the exact solution of Eq (5) and we therefore took a heuristic step-wise forward selection strategy to find a possibly suboptimal solution.

### Evaluation metrics and two-loop cross-validation

With the predictions of risk scores, we can evaluate prediction results based on an assessment of concordance index (CI) [71, 72], and an assessment of the statistical significance of separating censored survival data using a logrank test [73]. The CI is defined as the following:

$$\begin{array}{c} \text{CI}=\frac{\sum_{i,j}\delta_{i}\cdot 1(y_{j}>y_{i},\;\eta_{j}<\eta_{i})}{\sum_{i,j}\delta_{i}\cdot 1(y_{j}>y_{i})}, \end{array}$$ (6)

where 1(statement) is the indicator function. CI is a value similar to area under curve (AUC). A model reaches perfect prediction when CI = 1 and random guess when CI = 0.5. The logrank test uses a statistical test to accept or reject the null hypothesis $H_{0}$: two groups of samples share the same survival profile. It calculates a statistic *z*^2^ from observations of two groups of censored data. While $z\overset{d}{\to }N\left(0,1\right)$, we can get the *p*-value to accept or reject $H_{0}$. In our experiments, we split the samples in the test sets into two groups with the median of risk scores ${\left\{\eta_{i}\right\}}_{i=1}^{N}$, and used the logrank test to evaluate the differences between these two cohorts of predicted malignant and benign samples.

We used a two-loop cross-validation (CV) protocol to tune hyperparameters and evaluate the performance of different models, which consists of the inner CV and outer CV (Fig H in S1 Text). It is also referred to as “nested cross-validation” in the literature [74]. The inner CV is used for tuning parameters on the training set, i.e., λ in lasso model, *k* (and selected features) in the *ℓ*_0_-regularized model. The outer CV is used for **unbiased evaluation** on the test sets. This means that although the model may overfit at the inner CV time on the training sets due to larger model complexity or feature dimension, the final test sets at the outer CV are unseen to the model. By reporting the evaluation results only on the outer CV test sets, the evaluation protocol avoids the problem that a model with larger complexity tends to overfit and perform better through only inner CV. This is especially a concern when we compare the same model type with different features (Table 2 “clinical” vs. “full”), and when we compare the performance of different models such as *ℓ*_0_- and *ℓ*_1_-regularized Cox regression models (Table 2 vs. Table E in S1 Text). Note that we did not use a fixed training and test set split to replace the 3-fold outer CV. To some extent, the outer CV avoids the problem that test set may not be representative of the full data since we evaluated the full data during the outer CV.

### Tuning *ℓ*_0_-regularized Cox model: Subset selection

#### Heuristic subset selection: Step-wise forward feature selection

After extracting the three types of features, we aimed to utilize the *ℓ*_0_-regularized Cox regression for modeling and prediction. However, the hyperparameter *k* in the objective function Eq (5) is hard to tune. Given the $\beta \in R^{m}$, where *m* ranges between 100 and 200 in our case, we have to train and validate the 2^m^ models in a brute force way (or slightly faster using mixed integer linear programming [75]) to find the model with the optimal hyperparameter *k*, which is computationally intractable. Therefore, we propose using step-wise feature selection as a confrontation approach to finding suboptimal solutions in Eq (5). It is hard in our case to assess how well the forward selection heuristic approaches an optimal solution, since training and tuning the model optimally is a computationally intractable problem. However, a recent study on simulated data found step-wise forward selection performs only marginally worse than, if not as well as the optimal subset selection [76]. The validation tests of our method described below also suggest that it provides good solutions in practice. Careful readers may find the *ℓ*_0_-regularized problem will be slightly modified here if we use the heuristic step-wise feature selection method, because at the end of the step-wise feature selection through inner cross-validation (CV), we find/tune both the hyperparameter *k* and selected features, instead of just *k*. The selected features will be fixed at the time of evaluating the test set, not flexible.

In step-wise forward feature selection [77], we first traverse all the individual features in the candidate feature set, and evaluate the performance in concordance index (CI) of these features through the inner CV. The single feature with the best CV performance is selected. Secondly, we continue to traverse all the other unselected individual features in the candidate set, concatenate it with the first selected feature and evaluate the joint performance through the inner CV. The feature with the best performance is then taken as the second selected feature. We continue such a process to find the third, fourth, and fifth selected features until the inner CV performance does not increase. We implemented the step-wise forward selection of evolutionary, driver, clinical, genomic, and full feature sets using inner 3-fold CV in TCGA dataset and inner leave-one-out CV in ICGC dataset [71, 72]. We notice such a protocol is prone to overfit at the phase of inner CV compared with lasso. Other heuristics such as BIC may be appropriately revised and applied (though not trivial in Cox regression) to stop the forward steps early [78], which may potentially improve the model performance further by reducing the model complexity. However, even with the inner CV overfitting, *ℓ*_0_-regularized Cox model still outperforms a more conservative lasso Cox model on test sets of the outer CV (Table 2 vs. Table E in S1 Text).

#### Feature collinearity, bootstrapping, model interpretability and robustness

The correlations across features may lead to multicollinearity and fragile models, making the models hard to interpret. Prior to the step-wise forward selection tuning phase, we first randomly removed one of the features if any two features are highly correlated. We did not keep them both because technically the multicollinearity across features [75] could cause the numerical problems when taking the inverse of the covariance matrix, and lead to the lifelines optimizer failing to solve the problem. From the aspect of model performance, two features that are highly correlated capture similar information and it is less likely for the model to improve predictive power by taking both features as input. From the aspect of interpretability, the coefficients of the two correlated variables estimated from multivariable regression are unstable and inaccurate, making it hard to evaluate the importance of the feature. We used a Pearson coefficient threshold of 0.8 in absolute value to filter features, which is a commonly used threshold empirically [75]. Although a more carefully chosen threshold may potentially further improve the model performance, in practice, the bootstrapping (discussed below) is robust to the different thresholds in evaluating feature importance.

We noticed that such removal of features did not fully resolve the problem of model interpretability. For example, if both features are informative to the model but highly correlated, only one of the features will be kept. To better identify which individual features are predictive, we conducted bootstrapping. We drew 80% candidate features and 80% samples with replacement for 1,000 trials, and randomly selected one of the two features every time. The informative/essential features will be the ones selected frequently in bootstrapping. We refer readers to Sec. “Informative features for predicting tumor future progression” for two other approaches to evaluate feature importance in the context of feature collinearity, and their disadvantages compared with the bootstrapping.

Apart from the bootstrapping approach, there could be other solutions to address the collinearity problem, e.g., by pre-clustering the features into independent groups. However, the clustering method may also suffer in situations where one of the features can be represented as a complex linear combination of others, but no two features are highly correlated.

### Estimation of contribution from evolutionary features to progression risk

In multivariable regression, the different features may be intertwined in a complicated way. Therefore, the contributions of different features may be different when be placed together with other relevant features. We proposed a log-hazard-ratio-based fraction metric to calculate the fractions of contributions from evolutionary or genomic features among all the available data to the prognoses predictions [79]. The fraction metric normally lies within the range [0, 1], where zero means the evolutionary or genomic features do not contribute to the prediction at all, while one means the evolutionary or genomic features can explain all of the predictions even if other available features are provided. Theoretically, the fraction metric can be greater than one, which means the additional clinical or driver features are harmful to the model performance. However, empirically this does not happen in our experiments as clinical features are always helpful.

We define the log-hazard-ratio-based fraction of evolutionary features as below:

$$\begin{array}{c} \text{fraction}\left(\text{evolutionary}\right)=\frac{\log\text{HR}(\text{evolutionary})}{\log\text{HR}(\text{evolutionary+driver+clinical})}, \end{array}$$ (7)

where hazard ratio (HR) is defined as a value in [1, + ∞) when the feature set is informative of survival/recurrence:

$$\begin{array}{c} \text{HR}=\exp(-\sum_{i\in I_{M}}\beta^{⊺}X_{i})/\exp(-\sum_{i\in I_{B}}\beta^{⊺}X_{i}). \end{array}$$ (8)

where $I_{M}$ is the index set of samples predicted to be malignant (hazard larger than the median hazard), and $I_{B}$ is benign (hazard smaller than the median values).

We can use similar rules to define the contribution fractions of genomic features:

$$\begin{array}{c} \text{fraction}\left(\text{genomic}\right)=\frac{\log\text{HR}(\text{evolutionary+driver})}{\log\text{HR}(\text{evolutionary+driver+clinical})}, \end{array}$$ (9)

We use the log-scale HR instead of raw HR because the HR itself is calculated in the exponential scale of features. Taking the log allows us to directly compare the metric in the scale of the original feature space. Note that the fraction and CI are two different ways of evaluating the contribution and effectiveness of evolutionary features that would be expected to yield quantitatively different outcomes. The fraction aims to explain the feature contribution in the linear space of features, while CI aims to evaluate the effectiveness of features through performance. The calculation of fraction does not take into consideration unobservable variables, while the value of CI is affected by how much information is available in all the observed variables relative to unknown factors.

We calculated the HRs on the test sets at the outer CV phase for fraction estimation. The experiments were replicated five times to derive the mean HRs for the subsequent fraction estimation.

## Supporting information

S1 Text **Section A.1. Informative driver, clinical and full features for predicting tumor future progression**.
**Section B.1. Landscape of driver and clinical features**.
**Fig A. List of important features predictive of prognoses when evolutionary, driver, and clinical features are all available (TCGA dataset)**. We performed bootstrap replicates through cross-validation for 1,000 times, each time with 80% features and 80% samples drawn with replacement. The importance of features is quantified by the frequency with which they are selected. We show those features among the top 30 or selected for more than 50 times (frequency>0.05). See Fig B in S1 Text for important features when both evolutionary, driver, and clinical features were fed as candidate input at the same time in the ICGC dataset. See Figs 2 and 3 for important features in TCGA and ICGC dataset when single types of features are available.
**Fig B. List of important features predictive of prognoses when both evolutionary, driver, and clinical features are available (ICGC dataset)**. We performed bootstrap replicates through cross-validation for 1,000 times, each time with 80% features and 80% samples drawn with replacement. The importance of features is quantified by the frequency with which they are selected. We show those features among the top 30 or selected for more than 50 times (frequency>0.05). See Fig A in S1 Text for important features when both evolutionary, driver, and clinical features were fed as candidate input at the same time in the TCGA dataset. See Figs 2 and 3 for important features in TCGA and ICGC dataset when single types of features are available.
**Fig C. Pearson correlation for evolutionary, driver, and clinical features of LUCA samples**. (**A**) TCGA dataset, (**B**) ICGC dataset. See Fig 4 for feature correlation heatmaps of BRCA samples.
**Fig D. Manifolds in the evolutionary feature space are related to the future progression of BRCA and LUCA patients**. Figures are plotted based on samples in TCGA. We plotted the tSNE space of evolutionary features that shown to be important in the multivariate Cox regression (Fig 2). Each patient is represented as a single grey dot in the figure. The contours of survival time in the two-dimensional manifold are estimated based on the *k*-NN algorithm. We remove the area far from any of the sample points to avoid the artifacts generated from the *k*NN (e.g., small islands where there are no samples). The tumor samples lie in a manifold of the evolutionary feature space. There is a clear pattern that cancer patients in specific areas of the manifold have better or worse prognoses.
**Table A. Hazard ratios evaluated on the test sets**. We calculated the hazard ratio (HR) on the test sets of two-loop cross-validation following Eq (8), and repeated experiments for five times to calculate the mean and standard deviation of HRs. The results here are used to calculate the contribution fractions in Fig 5. See Table B in S1 Text for HRs when neoplasm status is removed from the clinical and full feature sets.
**Table B. Hazard ratios evaluated on the test sets when neoplasm status is removed from the clinical and full feature sets**. The results here are used to calculate the contribution fractions in Fig F in S1 Text.
**Table C. Contribution percentage of cumulative and phylogenetic evolutionary features to tumor progression risk prediction**. We estimated the fractions using Eqs (7) and (9) and data from Table A in S1 Text. Note the sum of contributions from cumulative and phylogenetic features is always larger than the contribution of evolutionary features in Fig 5. This is because the two types of evolutionary features are correlated and share part of the information, as shown in Fig 4 and Fig C in S1 Text.
**Fig E. Conditional distribution of death or recurrence rates given the neoplasm status clinical feature in BRCA and LUCA samples**. Patients with positive neoplasm status (*person neoplasm cancer status | tumor*) are much more prone to death or metastasis than tumor-free patients (*person neoplasm cancer status | tumor-free*), indicating that neoplasm status is a strong covariate for our regression model. The distribution is plotted using samples in TCGA.
**Fig F. Contribution percentage of evolutionary and genomic features to tumor progression risk prediction when neoplasm status is removed from the clinical features**. Evolutionary features contribute to around 50-60% in TCGA data, and 25-35% in ICGC data. The genomic features contribute to around 60-70% in TCGA, and 70-90% in ICGC dataset. We estimate the fractions using Eqs (7) and (9) and data from Table B in S1 Text. See Fig 5 for estimated fractions where neoplasm status is included in the clinincal features.
**Table D. Performance of prognostic prediction with different feature sets in TCGA and ICGC samples when the neoplasm status is removed from both the clinical and full feature sets**. We copy the results of “evolutionary”, “driver”, and “genomic” from Table 2 to facilitate comparison. One can observe similar performance to that in Table 2 where the tumor status is included.
**Table E. Performance of prognoses prediction using lasso (ℓ_1_-regularized Cox model) instead of *ℓ*_0_-regularized Cox regression model.**. The lasso model follows the same two-loop cross-validation evaluation protocol as *ℓ*_0_-regularized model, and replicates for five times to get the mean and standard deviation values of performance in concordance index (CI). The cell background is pink if lasso performs better than *ℓ*_0_-regularized model; it is blue if lasso performs worse; white background means there is no significant difference between the two models. One can find that *ℓ*_0_-regularized Cox model performs better than lasso in most cases (29/40), while lasso performs better only in 6 cases.
**Fig G. Top selected evolutionary features when the relations between features and prognoses are broken (TCGA dataset)**. We permutated the evolutionary features across samples, and followed the bootstrapping protocols in Fig 2. The top 10 most frequently selected evolutionary features are evenly distributed, and lose the informative pattern in Fig 2. This indicates the original evolutionary features are not just random noises or artifacts.
**Table F. Performance of prognostic prediction with shuffled features in WES-based TCGA and WGS-based ICGC samples**. All the feature types are randomly permutated across samples. Then we followed the same experimental protocols in Table 2 to evaluate the prognostic prediction performance with these shuffled features. The performance is almost random as evaluated by the CI (around 50%). In addition, the permutation test exhibits a much larger variance in prediction results compared with the raw data in Table 2.
**Section H. Additional details on use of Canopy and TUSV**.
**Section I. Feature extraction and preprocessing**. **Table G. List of cumulative evolutionary features**. The mutation rates related to SNVs, CNAs and SVs of samples are included. All cumulative evolutionary features are in continuous value. We have 6 mutation rates and 96 trinucleotide mutation rates as features in total.
**Table H. List of phylogenetic evolutionary features**. Due to the different output of Canopy (phylogenetic model for WES) and TUSV (phylogenetic model for WGS), the sets of phylogenetic features are slightly different. The WGS data contain additional features related to CNA and SV rates.
**Table I. List of driver features**. The potential drivers come from both IntOGen and COSMIC databases. BRCA and LUCA share a large portion of drivers. We count the somatic mutation rates of both SNVs, indels, CNAs, and SVs in all drivers as the driver features. These features are in continuous value.
**Table J. List of clinical features**. BRCA and LUCA samples share a large portion of similar clinical features. Three data types are available: binary, categorical and continuous. Cancer subtype is denoted as *histological type*.
**Fig H. Procedure of training, tuning, and unbiased evaluation through two-loop cross-validation**. The whole dataset is split into three parts and evaluated through 3-fold outer cross-validation (CV) on the test sets. In each experiment of the outer CV, the *ℓ*_1_- or *ℓ*_0_-regularized model is tuned using an inner CV only on the training set. We used 3-fold inner CV for TCGA and leave-one-out inner CV (LOOCV) for ICGC throughout the work. We employed LOOCV for ICGC because it has a much smaller sample size. The utilization of two-loop CV prevents the problem of bias when we evaluate models with different complexities, e.g., Cox model using clinical features vs. Cox model using both clinical and genomic features, or *ℓ*_1_-regularized model vs. *ℓ*_0_-regularized model. In contrast, the model with larger complexity tends to perform “better” due to overfitting if using the single-loop CV.
(PDF)
