# Supplementary material for: Assessing the contribution of tumor mutational phenotypes to cancer progression risk
Source: PLoS Comput Biol. 2021 Mar 12;17(3):e1008777. doi: 10.1371/journal.pcbi.1008777 (PMC7990181; doi:10.1371/journal.pcbi.1008777)
Supplement: S1 Text — Section A.1. Informative driver, clinical and full features for predicting tumor future progression. Section B.1. Landscape of driver and clinical features. Fig A. List of important features predictive of prognoses when evolutionary, driver, and clinical features are all available (TCGA dataset). We performed bootstrap replicates through cross-validation for 1,000 times, each time with 80% features and 80% samples drawn with replacement. The importance of features is quantified by the frequency with which they are selected. We show those features among the top 30 or selected for more than 50 times (frequency>0.05). See Fig B in S1 Text for important features when both evolutionary, driver, and clinical features were fed as candidate input at the same time in the ICGC dataset. See Figs 2 and 3 for important features in TCGA and ICGC dataset when single types of features are available. Fig B. List of important features predictive of prognoses when both evolutionary, driver, and clinical features are available (ICGC dataset). We performed bootstrap replicates through cross-validation for 1,000 times, each time with 80% features and 80% samples drawn with replacement. The importance of features is quantified by the frequency with which they are selected. We show those features among the top 30 or selected for more than 50 times (frequency>0.05). See Fig A in S1 Text for important features when both evolutionary, driver, and clinical features were fed as candidate input at the same time in the TCGA dataset. See Figs 2 and 3 for important features in TCGA and ICGC dataset when single types of features are available. Fig C. Pearson correlation for evolutionary, driver, and clinical features of LUCA samples. (A) TCGA dataset, (B) ICGC dataset. See Fig 4 for feature correlation heatmaps of BRCA samples. Fig D. Manifolds in the evolutionary feature space are related to the future progression of BRCA and LUCA patients. Figures are plotted based on samples in TCGA. We plotted t [file pcbi.1008777.s001.pdf]

Supporting information for “Assessing the contribution of tumor mutational phenotypes to cancer progression risk”

Section A.1 in S1 Text. Informative driver, clinical and full features for predicting tumor future progression.

Section B.1 in S1 Text. Landscape of driver and clinical features.

Fig A in S1 Text. List of important features predictive of prognoses when evolutionary, driver, and clinical features are all available (TCGA dataset). We performed bootstrap replicates through cross-validation for 1,000 times, each time with 80% features and 80% samples drawn with replacement. The importance of features is quantified by the frequency with which they are selected. We show those features among the top 30 or selected for more than 50 times (frequency>0.05). See Fig B in S1 Text for important features when both evolutionary, driver, and clinical features were fed as candidate input at the same time in the ICGC dataset. See Fig 2,3 for important features in TCGA and ICGC dataset when single types of features are available.

Fig B in S1 Text. List of important features predictive of prognoses when both evolutionary, driver, and clinical features are available (ICGC dataset). We performed bootstrap replicates through cross-validation for 1,000 times, each time with 80% features and 80% samples drawn with replacement. The importance of features is quantified by the frequency with which they are selected. We show those features among the top 30 or selected for more than 50 times (frequency>0.05). See Fig A in S1 Text for important features when both evolutionary, driver, and clinical features were fed as candidate input at the same time in the TCGA dataset. See Fig 2,3 for important features in TCGA and ICGC dataset when single types of features are available.

Fig C in S1 Text. Pearson correlation for evolutionary, driver, and clinical features of LUCA samples. (**A**) TCGA dataset, (**B**) ICGC dataset. See Fig 4 for feature correlation heatmaps of BRCA samples.

Fig D in S1 Text. Manifolds in the evolutionary feature space are related to the future progression of BRCA and LUCA patients. Figures are plotted based on samples in TCGA. We plotted the tSNE space of evolutionary features that shown to be important in the multivariate Cox regression (Fig 2). Each patient is represented as a single grey dot in the figure. The contours of survival time in the two-dimensional manifold are estimated based on the k-NN algorithm. We remove the area far from any of the sample points to avoid the artifacts generated from the kNN (e.g., small islands where there are no samples). The tumor samples lie in a manifold of the evolutionary feature space. There is a clear pattern that cancer patients in specific areas of the manifold have better or worse prognoses.

Table A in S1 Text. Hazard ratios evaluated on the test sets. We calculated the hazard ratio (HR) on the test sets of two-loop cross-validation following Eq (8), and repeated experiments for five times to calculate the mean and standard deviation of HRs. The results here are used to calculate the contribution fractions in Fig 5. See Table B in S1 Text for HRs when neoplasm status is removed from the clinical and full feature sets.

Table B in S1 Text. Hazard ratios evaluated on the test sets when neoplasm status is removed from the clinical and full feature sets. The results here are used to calculate the contribution fractions in Fig F in S1 Text.

Table C in S1 Text. Contribution percentage of cumulative and phylogenetic evolutionary features to tumor progression risk prediction. We estimated the fractions using Eq (7,9) and data from Table A in S1 Text. Note the sum of contributions from cumulative and phylogenetic features is always larger than the contribution of evolutionary features in Fig 5. This is because the two types of evolutionary features are correlated and share part of the information, as shown in Fig 4 and Fig C in S1 Text.

Fig E in S1 Text. Conditional distribution of death or recurrence rates given the neoplasm status clinical feature in BRCA and LUCA samples. Patients with positive neoplasm status (*person neoplasm cancer status* | *tumor*) are much more prone to death or metastasis than tumor-free patients (*person neoplasm cancer status* | *tumor-free*), indicating that neoplasm status is a strong covariate for our regression model. The distribution is plotted using samples in TCGA.

Fig F in S1 Text. Contribution percentage of evolutionary and genomic features to tumor progression risk prediction when neoplasm status is removed from the clinical features. Evolutionary features contribute to around 50-60% in TCGA data, and 25-35% in ICGC data. The genomic features contribute to around 60-70% in TCGA, and 70-90% in ICGC dataset. We estimate the fractions using Eq (7,9) and data from Table B in S1 Text. See Fig 5 for estimated fractions where neoplasm status is included in the clinincal features.

Table D in S1 Text. Performance of prognostic prediction with different feature sets in TCGA and ICGC samples when the neoplasm status is removed from both the clinical and full feature sets. We copy the results of "evolutionary", "driver", and "genomic" from Table 2 to facilitate comparison. One can observe similar performance to that in Table 2 where the tumor status is included.

Table E in S1 Text. Performance of prognoses prediction using lasso (ℓ_1-regularized Cox model) instead of ℓ_0-regularized Cox regression model. The lasso model follows the same two-loop cross-validation evaluation protocol as ℓ_0-regularized model, and replicates for five times to get the mean and standard deviation values of performance in concordance index (CI). The cell background is pink if lasso performs better than ℓ_0-regularized model; it is blue if lasso performs worse; white background means there is no significant difference between the two models. One can find that ℓ_0-regularized Cox model performs better than lasso in most cases (29/40), while lasso performs better only in 6 cases.

Fig G in S1 Text. Top selected evolutionary features when the relations between features and prognoses are broken (TCGA dataset). We permutated the evolutionary features across samples, and followed the bootstrapping protocols in Fig 2. The top 10 most frequently selected evolutionary features are evenly distributed, and lose the informative pattern in Fig 2. This indicates the original evolutionary features are not just random noises or artifacts.

Table F in S1 Text. Performance of prognostic prediction with shuffled features in WES-based TCGA and WGS-based ICGC samples. All the feature types are randomly permutated across samples. Then we followed the same experimental protocols in Table 2 to evaluate the prognostic prediction performance with these shuffled features. The performance is almost random as evaluated by the CI (around 50%). In addition, the permutation test exhibits a much larger variance in prediction results compared with the raw data in Table 2.

Section H in S1 Text. Additional details on use of Canopy and TUSV.

Section I in S1 Text. Feature extraction and preprocessing.

Table G in S1 Text. List of cumulative evolutionary features. The mutation rates related to SNVs, CNAs and SVs of samples are included. All cumulative evolutionary features are in continuous value. We have 6 mutation rates and 96 trinucleotide mutation rates as features in total.

Table H in S1 Text. List of phylogenetic evolutionary features. Due to the different output of Canopy (phylogenetic model for WES) and TUSV (phylogenetic model for WGS), the sets of phylogenetic features are slightly different. The WGS data contain additional features related to CNA and SV rates.

Table I in S1 Text. List of driver features. The potential drivers come from both IntOGen and COSMIC databases. BRCA and LUCA share a large portion of drivers. We count the somatic mutation rates of both SNVs, indels, CNAs, and SVs in all drivers as the driver features. These features are in continuous value.

Table J in S1 Text. List of clinical features. BRCA and LUCA samples share a large portion of similar clinical features. Three data types are available: binary, categorical and continuous. Cancer subtype is denoted as *histological type*.

Fig H in S1 Text. Procedure of training, tuning, and unbiased evaluation through two-loop cross-validation. The whole dataset is split into three parts and evaluated through 3-fold outer cross-validation (CV) on the test sets. In each experiment of the outer CV, the ℓ_1- or ℓ_0-regularized model is tuned using an inner CV only on the training set. We used 3-fold inner CV for TCGA and leave-one-out inner CV (LOOCV) for ICGC throughout the work. We employed LOOCV for ICGC because it has a much smaller sample size. The utilization of two-loop CV prevents the problem of bias when we evaluate models with different complexities, e.g., Cox model using clinical features vs. Cox model using both clinical and genomic features, or ℓ_1-regularized model vs. ℓ_0-regularized model. In contrast, the model with larger complexity tends to perform "better" due to overfitting if using the single-loop CV.

A Informative features

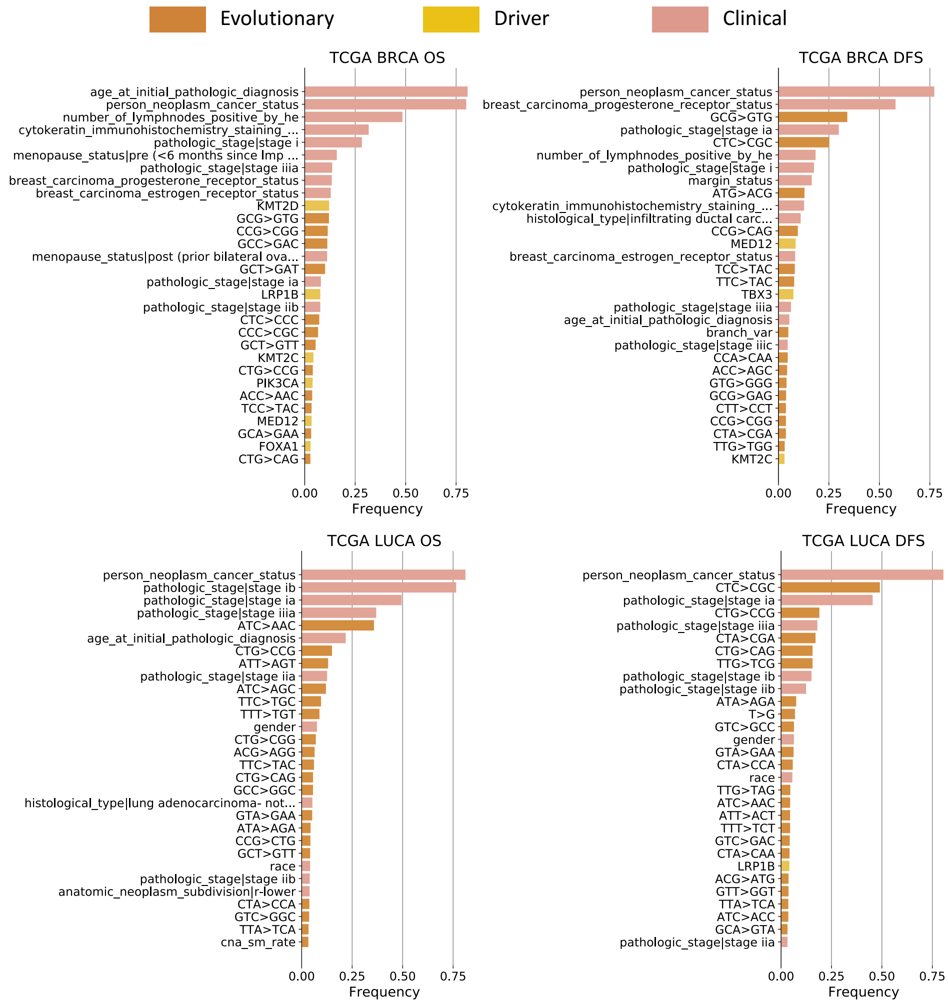

Fig A. List of important features predictive of prognoses when evolutionary, driver, and clinical features are all available (TCGA dataset). We performed bootstrap replicates through cross-validation for 1,000 times, each time with 80% features and 80% samples drawn with replacement. The importance of features is quantified by the frequency with which they are selected. We show those features among the top 30 or selected for more than 50 times (frequency>0.05). See Fig for important features when both evolutionary, driver, and clinical features were fed as candidate input at the same time in the ICGC dataset. See Fig 2,3 for important features in TCGA and ICGC dataset when single types of features are available.

A.1 Informative driver, clinical and full features for predicting tumor future progression

Driver features A few driver features, such as *TBX3* and *MED12*, are informative for both OS and DFS prediction in breast cancer (Fig 2,3 BRCA driver bars) [1,2]. Some of the most well-established breast driver genes with fairly high population frequencies are not selected with high frequency in all analyses (e.g., *ERBB2* does not

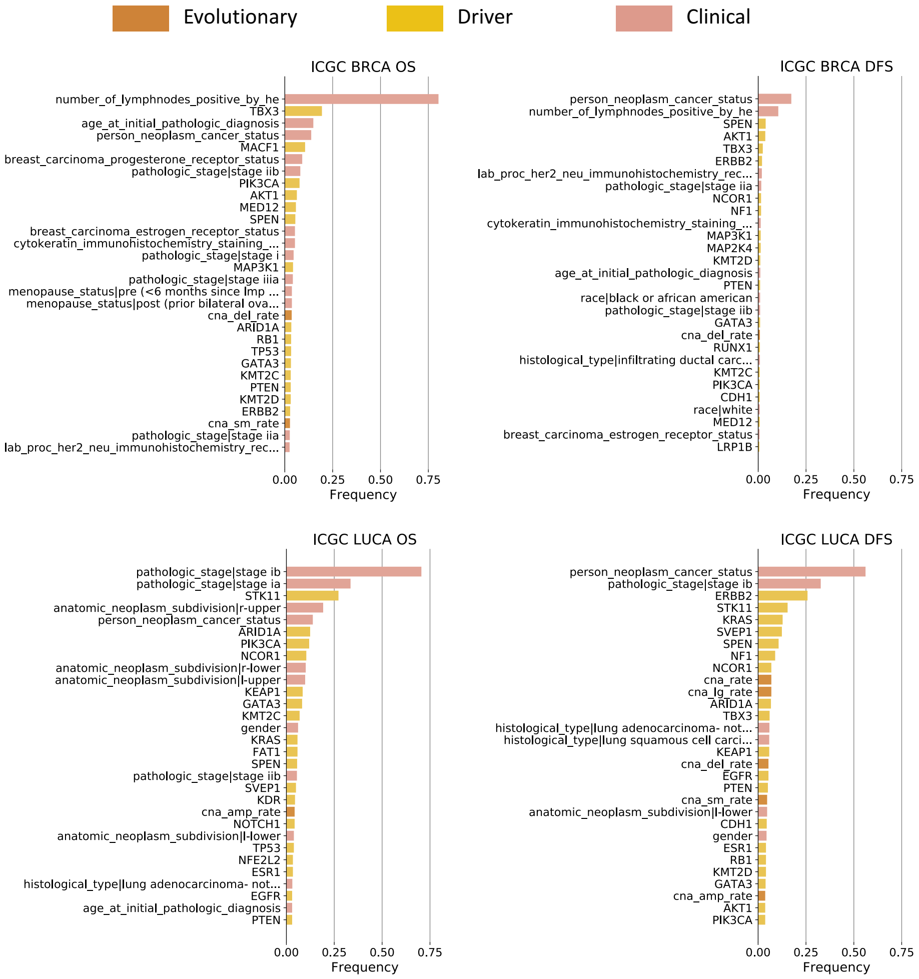

Fig B. List of important features predictive of prognoses when both evolutionary, driver, and clinical features are available (ICGC dataset). We performed bootstrap replicates through cross-validation for 1,000 times, each time with 80% features and 80% samples drawn with replacement. The importance of features is quantified by the frequency with which they are selected. We show those features among the top 30 or selected for more than 50 times (frequency>0.05). See Fig for important features when both evolutionary, driver, and clinical features were fed as candidate input at the same time in the TCGA dataset. See Fig 2,3 for important features in TCGA and ICGC dataset when single types of features are available.

appear in the ICGC OS list while *TP53* does not appear in the ICGC DFS list), which likely reflects at least in part the fact that other features can serve as proxies for these and thus they are not consistently chosen by the machine learning feature selection.

The driver feature *LRP1B* is the most informative for both prediction tasks in lung cancer (Fig 2,3 LUCA driver bars) [3]. We observe a big difference of selected drivers in the LUCA samples between TCGA and ICGC datasets, though. This is mainly caused by the sparsity of the driver mutation rates in WES-based TCGA data. Most of the drivers are mutated at less than 5% in frequency (zero values in more than 95% samples) and are therefore excluded at the time of feature engineering.

Clinical features Tumor neoplasm status (*person neoplasm cancer status*), *pathologic stage*, *age at initial pathologic diagnosis*, and cancer subtype (*histological type*) emerge as the top clinical features across the eight analyses (Fig 2,3 clinical bars). The neoplasm status reports whether the patient has a tumor or is tumor-free. The high frequency of inclusion of pathologic stage and subtype in successful predictors reflects the known value of expert staging and classification in predicting cancer outcome [4], and their inclusion is thus also unsurprising. The importance of age may in part reflect the cancer-intrinsic effects of somatic mutation processes that are likely to have been active longer in tumors of older versus younger patients. However, older patients are more likely to die of other competing risks, such as heart attack, than their cancer during the time of follow-up [5]. Teasing apart the various confounding factors introduced by age at diagnosis is a complicated question, however, beyond the scope of the present study [6].

OS and DFS prediction tasks extensively share clinical features within the same cancer type. For example, PR status (*progesterone receptor status*) and ER status (*estrogen receptor status*) are highly informative for both tasks in BRCA, which is consistent with the current subtype classification method of breast cancer based on hormone receptor status [7]. The clinical features *number of lymphnodes positive by he* and *cytokeratin immunohistochemistry staining method micrometastasis indicator* are consistent with previous research on the value of these factors as predictors of breast cancer survival [8] and metastasis [9]. While in the present work, we directly take cytokeratin immunohistochemistry staining as a feature, one should note the possible artifacts that could be introduced in practice [10]. In lung cancer, both the clinical features *anatomic neoplasm subdivision* and *gender* are predictive for both OS and DFS, consistent with the previous research that the right lung and male gender are usually associated with worse prognoses [11]. The male and female samples are balanced (53% vs. 47%) in the dataset.

Full feature sets The combinations of features that lead to the best performance in TCGA and ICGC when all feature classes are treated collectively during bootstrapping are collected in Fig A in S1 Text and Fig B in S1 Text. In all cases, the high-ranking features in the full feature sets include mixtures of evolutionary, driver, and clinical features. For the most part, the selected features that appear in the full feature bootstrapping are subsets of those found when bootstrapping on individual feature sets. This indicates that the features from the three sets in general provide at least some orthogonal information to one another, and all contribute to the prediction of prognoses. While the top-ranking features in the collective sets are usually drawn from the clinical feature set, the frequencies of many clinical features decrease and some drop out of the list in the full experiments. This observation indicates that the effectiveness of these features diminishes and they can become redundant when some useful genomic features are included. The importance order of features is also frequently changed in the full feature sets, again suggesting correlations between features from distinct classes. Some genomic features are not selected as predictive when combined only with other genomic features, but proved predictive in combination with clinical features, e.g., $ATG \rightarrow ACG$ in the TCGA BRCA DFS task (Fig A in S1 Text).

B Feature landscape

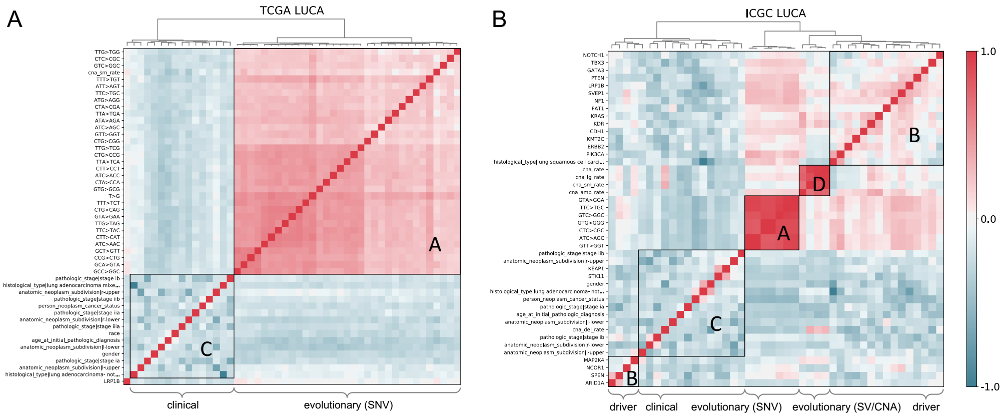

Fig C. Pearson correlation for evolutionary, driver, and clinical features of LUCA samples. (**a**) TCGA dataset, (**b**) ICGC dataset. See Fig 4 for feature correlation heatmaps of BRCA samples.

B.1 Landscape of driver and clinical features

Driver features (block-B): Driver features mostly form a distinct block of high mutual correlation. This block is moderately correlated with both classes of evolutionary rate features. We hypothesize that the general pattern of positive correlation among driver features reflects generic differences in background SNV or CNA rates from different somatic hypermutability phenotypes [12]. For example, we propose that tumors exhibiting one CNA driver mutation are more likely to exhibit other CNA driver mutations because they are more likely to have an elevated rate of CNA mutations generically. This interpretation is confirmed by the positive cross-correlations between driver features and CNA rate features (Fig 4b) and between driver and SNV rate features (Fig 4a,b). **Clinical features (block-C):** Most clinical features are negatively, rather than positively, correlated with other clinical features outside of small, highly correlated sub-blocks. The main reason comes from the data processing and feature extraction step when we map categorical clinical features into one-hot vectors and therefore introduce collinearity. For example, the *menopause status | pre* and *menopause status | post* originally came from the same categorical clinical feature *menopause status* to yield two anti-correlated binary features. We can break this type of collinearity while maintaining the model interpretability by dropping one of the anti-correlated categories and bootstrapping. Strong positive correlation is also sometimes observed within clinical features for biological reasons, such as that between PR status (*progesterone receptor status*) and ER status (*estrogen receptor status*) reflecting a common association with luminal breast cancer subtypes [7], or that between *age* and *menopause status*.

B.2 Manifolds of evolutionary features

We sought to explore aspects of the correlation structure that might not be readily apparent solely from the pairwise correlation analysis of evolutionary features. To investigate the landscape of tumors in the evolutionary feature space, we considered all the evolutionary features that were shown to be important to prognoses (Fig 2 TCGA OS/DFS evolutionary bars). We applied this analysis only to the TCGA dataset instead of ICGC because of its $\sim$10 times larger sample size, which enables us to reliably infer the manifolds and effectively reduce the noise during analysis. In order to visualize the manifold defined by cancer patients in this space of evolutionary features, we conducted tSNE to reduce the sample points to two dimensions (Fig). We represented each sample as a grey dot, and interpolated survival or recurrence time in the feature space using k-nearest neighbors (k-NN) on deceased samples to reduce the noise from survival data of individual samples. k was chosen to be 16, which is large enough to smooth the estimated OS/DFS, while small enough to keep enough details in the feature space. We fitted the contours using geometric mean instead of arithmetic mean of the OS/DFS since they both have long-tail distributions. The darker color represents shorter estimated survival/recurrence time and thus more malignant status, and vice versa. There exist local artifacts of the contours using k-NN, when k is larger than one, e.g., the small lighter/darker islands the area where there are no points. We masked space that is far from any sample points to alleviate such artifacts visually.

As the figures show (Fig), most of the patients roughly locate in a (nearly) continuous low dimensional manifold. There exist patterns and internal structures of OS or DFS time for both BRCA and LUCA patients in the tSNE-reduced manifolds. The future survival time and recurrence time are related to the locations of tumor samples on the manifolds. That is, there exist both specific lighter areas in BRCA OS/DFS, that are indicative of optimistic prognoses and benign tumor future progression. Similar darker areas exist in LUCA OS/DFS, which indicate regions in feature space corresponding to poor prognoses and malignant tumor future progression.

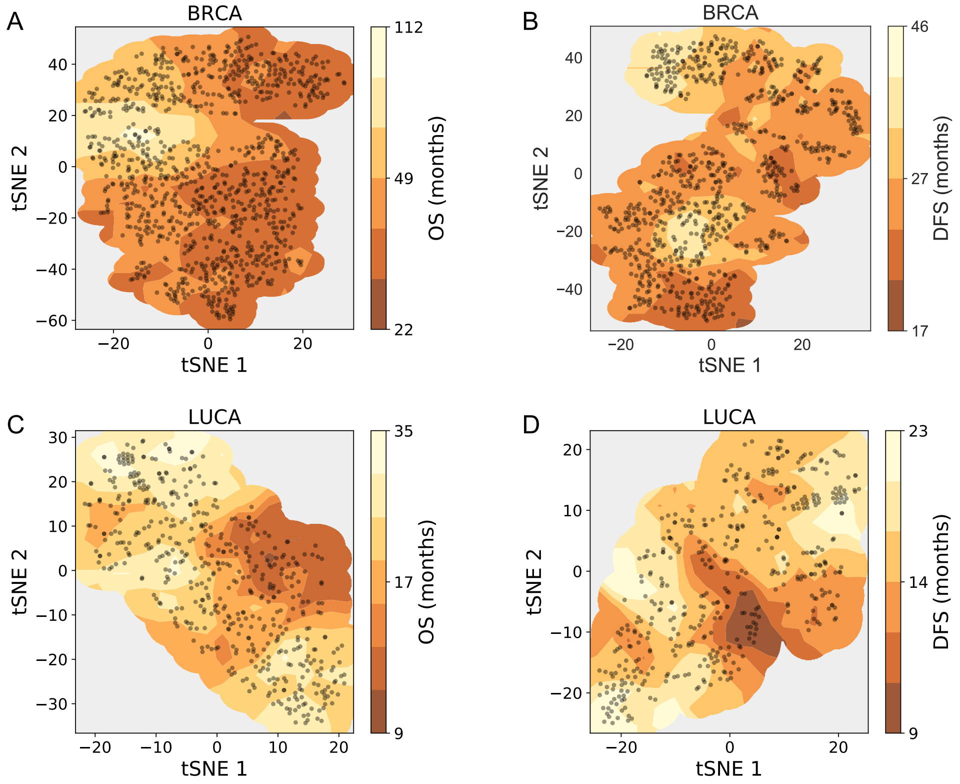

Fig D. Manifolds in the evolutionary feature space are related to the future progression of BRCA and LUCA patients. Figures are plotted based on samples in TCGA. We plotted the tSNE space of evolutionary features that shown to be important in the multivariate Cox regression (Fig 2). Each patient is represented as a single grey dot in the figure. The contours of survival time in the two-dimensional manifold are estimated based on the k-NN algorithm. We remove the area far from any of the sample points to avoid the artifacts generated from the kNN (e.g., small islands where there are no samples). The tumor samples lie in a manifold of the evolutionary feature space. There is a clear pattern that cancer patients in specific areas of the manifold have better or worse prognoses.

C Hazard ratios

	TCGA (WES)				ICGC (WGS)			
	BRCA		LUCA		BRCA		LUCA	
HR	OS	DFS	OS	DFS	OS	DFS	OS	DFS
cumulative	1.26±0.008	1.22±0.007	1.31±0.013	1.27±0.014	1.19±0.018	1.25±0.017	1.27±0.023	1.35±0.017
phylogenetic	1.12±0.004	1.07±0.006	1.10±0.008	1.09±0.004	1.19±0.013	1.17±0.010	1.18±0.022	1.17±0.020
evolutionary	1.28±0.005	1.23±0.007	1.34±0.007	1.30±0.009	1.23±0.022	1.32±0.017	1.32±0.020	1.40±0.018
driver	1.18±0.004	1.21±0.006	1.28±0.001	1.22±0.001	1.64±0.032	1.64±0.041	1.86±0.037	1.95±0.050
genomic	1.32±0.011	1.30±0.011	1.40±0.015	1.35±0.022	1.84±0.051	1.90±0.106	2.06±0.068	2.28±0.097
clinical	1.70±0.014	1.56±0.011	1.94±0.014	1.86±0.035	2.15±0.040	2.26±0.045	2.04±0.063	2.08±0.056
full	1.76±0.009	1.61±0.011	2.03±0.020	1.94±0.016	2.46±0.055	2.47±0.079	2.64±0.072	2.65±0.052

Table A. Hazard ratios evaluated on the test sets. We calculated the hazard ratio (HR) on the test sets of two-loop cross-validation following Eq (8), and repeated experiments for five times to calculate the mean and standard deviation of HRs. The results here are used to calculate the contribution fractions in Fig 5 and Table . See Table B for HRs when neoplasm status is removed from the clinical and full feature sets.

	TCGA (WES)				ICGC (WGS)			
	BRCA		LUCA		BRCA		LUCA	
HR	OS	DFS	OS	DFS	OS	DFS	OS	DFS
cumulative	1.26±0.008	1.22±0.007	1.31±0.013	1.27±0.014	1.19±0.018	1.25±0.017	1.27±0.023	1.35±0.017
phylogenetic	1.12±0.004	1.07±0.006	1.10±0.008	1.09±0.004	1.19±0.013	1.17±0.010	1.18±0.022	1.17±0.020
evolutionary	1.28±0.005	1.23±0.007	1.34±0.007	1.30±0.009	1.23±0.022	1.32±0.017	1.32±0.020	1.40±0.018
driver	1.18±0.004	1.21±0.006	1.28±0.001	1.22±0.001	1.64±0.032	1.64±0.041	1.86±0.037	1.95±0.050
genomic	1.32±0.011	1.30±0.011	1.40±0.015	1.35±0.022	1.84±0.051	1.90±0.106	2.06±0.068	2.28±0.097
clinicalΔ	1.52±0.014	1.42±0.017	1.58±0.015	1.37±0.014	2.06±0.042	2.10±0.061	1.91±0.035	1.81±0.043
fullΔ	1.61±0.010	1.52±0.016	1.71±0.023	1.51±0.020	2.45±0.056	2.46±0.058	2.57±0.051	2.52±0.052

Table B. Hazard ratios evaluated on the test sets when neoplasm status is removed from the clinical and full feature sets. The results here are used to calculate the contribution fractions in Fig F in S1 Text.

D Risk contribution of cumulative and phylogenetic evolutionary features

	TCGA (WES)				ICGC (WGS)			
	BRCA		LUCA		BRCA		LUCA	
**Fraction (%)**	**OS**	**DFS**	**OS**	**DFS**	**OS**	**DFS**	**OS**	**DFS**
cumulative	41.4	41.5	38.6	36.3	19.5	25.2	24.4	31.0
phylogenetic	19.3	14.2	13.5	13.4	19.7	17.2	17.2	16.0

Table C. Contribution percentage of cumulative and phylogenetic evolutionary features to tumor progression risk prediction. We estimated the fractions using Eq (7,9) and data from Table A in S1 Text. Note the sum of contributions from cumulative and phylogenetic features is always larger than the contribution of evolutionary features in Fig 5. This is because the two types of evolutionary features are correlated and share part of the information, as shown in Fig 4 and Fig C in S1 Text.

E Results with neoplasm status excluded

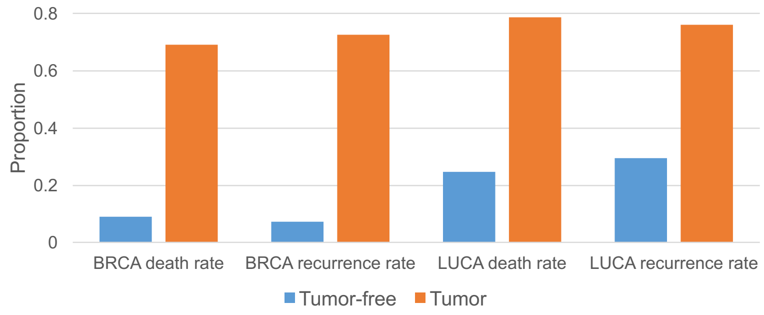

Fig E. Conditional distribution of death or recurrence rates given the neoplasm status clinical feature in BRCA and LUCA samples. Patients with positive neoplasm status (*person neoplasm cancer status* | *tumor*) are much more prone to death or metastasis than tumor-free patients (*person neoplasm cancer status* | *tumor-free*), indicating that neoplasm status is a strong covariate for our regression model. The distribution is plotted using samples in TCGA.

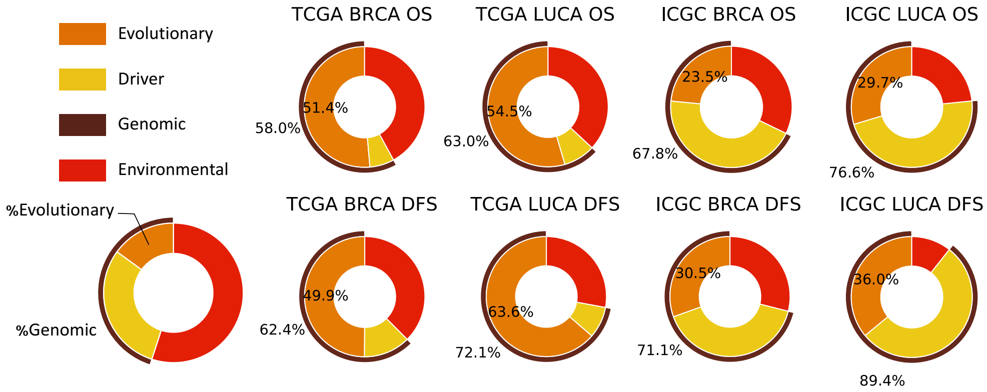

Fig F. Contribution percentage of evolutionary and genomic features to tumor progression risk prediction when neoplasm status is removed from the clinical features. Evolutionary features contribute to around 50-60% in TCGA data, and 25-35% in ICGC data. The genomic features contribute to around 60-70% in TCGA, and 70-90% in ICGC dataset. We estimate the fractions using Eq. (7,9) and data from Table . See Fig 5 for estimated fractions where neoplasm status is included in the clinincal features.

	TCGA (WES)				ICGC (WGS)			
	BRCA		LUCA		BRCA		LUCA	
**CI**	**OS**	**DFS**	**OS**	**DFS**	**OS**	**DFS**	**OS**	**DFS**
evolutionary	56.9$\pm$0.56	53.3$\pm$0.44	51.8$\pm$0.26	50.5$\pm$0.25	51.7$\pm$0.74	54.0$\pm$1.21	52.9$\pm$0.73	53.2$\pm$0.66
driver	54.9$\pm$0.54	55.4$\pm$0.59	53.6$\pm$0.05	53.6$\pm$0.03	53.1$\pm$0.97	51.1$\pm$0.46	51.2$\pm$0.59	50.4$\pm$0.59
genomic	59.2$\pm$0.49	56.2$\pm$1.01	53.2$\pm$0.35	51.7$\pm$0.38	57.5$\pm$2.15	56.2$\pm$2.30	52.8$\pm$1.28	54.5$\pm$1.03
clinicalΔ	68.9$\pm$0.64	63.4$\pm$0.76	63.7$\pm$0.48	55.9$\pm$0.82	75.1$\pm$1.37	69.9$\pm$1.30	**61.9**$\pm$1.81^{ns}	52.4$\pm$0.74
fullΔ	**71.3**$\pm$0.57***	**64.6**$\pm$0.85*	**64.1**$\pm$0.46^{ns}	**56.4**$\pm$0.65^{ns}	**81.1**$\pm$1.52***	**72.2**$\pm$2.41$^{\cdot}$	61.3$\pm$1.31	**55.6**$\pm$1.62**

Table D. Performance of prognostic prediction with different feature sets in TCGA and ICGC samples when the neoplasm status is removed from both the clinical and full feature sets. We copy the results of "evolutionary", "driver", and "genomic" from Table 2 to facilitate comparison. One can observe similar performance to that in Table 2 where the tumor status is included.

F Performance comparison of lasso and ℓ_0-regularized Cox models

	TCGA (WES)				ICGC (WGS)			
	BRCA		LUCA		BRCA		LUCA	
CI	OS	DFS	OS	DFS	OS	DFS	OS	DFS
evolutionary	58.0±0.12	52.8±0.39	50.8±0.26	50.4±0.15	51.0±0.58	53.9±1.03	52.8±0.59	50.4±0.50
driver	55.3±0.40	55.1±0.57	53.6±0.06	53.6±0.03	52.7±1.44	51.1±0.74	50.0±0.08	50.1±0.21
genomic	58.0±0.19	54.4±0.30	51.6±0.41	50.6±0.19	51.1±0.71	54.2±0.90	52.2±0.45	50.5±0.43
clinical	78.2±0.47	74.8±0.52	67.6±0.72	63.1±0.55	70.9±3.91	73.7±4.02	62.1±1.55	55.6±2.07
full	79.6±0.19	72.6±0.34	67.5±0.47	64.5±0.42	77.9±1.76	69.5±3.50	57.3±1.15	58.4±1.79

Table E. Performance of prognoses prediction using lasso (ℓ_1-regularized Cox model) instead of ℓ_0-regularized Cox regression model. The lasso model follows the same two-loop cross-validation evaluation protocol as ℓ_0-regularized model, and replicates for five times to get the mean and standard deviation values of performance in concordance index (CI). The cell background is pink if lasso performs better than ℓ_0-regularized model; it is blue if lasso performs worse; white background means there is no significant difference between the two models. One can find that ℓ_0-regularized Cox model performs better than lasso in most cases (29/40), while lasso performs better only in 6 cases.

G Permutation tests

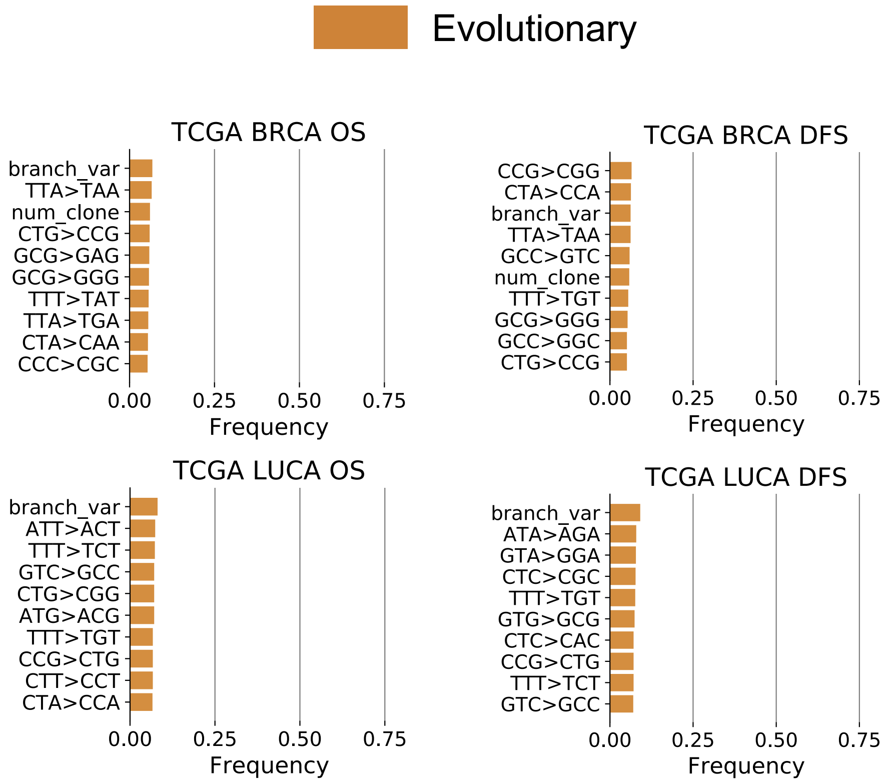

Fig G. Top selected evolutionary features when the relations between features and prognoses are broken (TCGA dataset). We permutated the evolutionary features across samples, and followed the bootstrapping protocols in Fig 2. The top 10 most frequently selected evolutionary features are evenly distributed, and lose the informative pattern in Fig 2. This indicates the original evolutionary features are not just random noises or artifacts.

Table F. Performance of prognostic prediction with shuffled features in WES-based TCGA and WGS-based ICGC samples.

	TCGA (WES)				ICGC (WGS)			
	BRCA		LUCA		BRCA		LUCA	
**CI**	**OS**	**DFS**	**OS**	**DFS**	**OS**	**DFS**	**OS**	**DFS**
evolutionary	$50.1_{\pm 3.68}$	$50.5_{\pm 4.72}$	$49.9_{\pm 1.60}$	$51.4_{\pm 2.01}$	$48.2_{\pm 8.01}$	$51.8_{\pm 8.76}$	$46.6_{\pm 8.73}$	$50.3_{\pm 7.58}$
driver	$53.1_{\pm 2.42}$	$47.8_{\pm 3.30}$	$50.5_{\pm 2.70}$	$50.3_{\pm 1.36}$	$42.3_{\pm 7.45}$	$47.6_{\pm 11.46}$	$51.6_{\pm 3.44}$	$50.2_{\pm 7.65}$
genomic	$52.0_{\pm 3.12}$	$46.1_{\pm 4.84}$	$49.3_{\pm 2.61}$	$49.4_{\pm 2.13}$	$43.9_{\pm 12.26}$	$48.0_{\pm 9.46}$	$46.2_{\pm 6.80}$	$43.9_{\pm 4.35}$
clinical	$48.2_{\pm 4.12}$	$47.3_{\pm 3.33}$	$50.5_{\pm 3.58}$	$48.2_{\pm 2.73}$	$44.0_{\pm 9.17}$	$49.1_{\pm 12.61}$	$45.5_{\pm 5.41}$	$52.1_{\pm 8.48}$
full	$51.0_{\pm 3.00}$	$48.3_{\pm 5.65}$	$49.0_{\pm 1.75}$	$50.3_{\pm 2.63}$	$54.2_{\pm 5.97}$	$50.8_{\pm 8.30}$	$51.0_{\pm 4.75}$	$44.5_{\pm 4.66}$

All feature types are randomly permuted across samples. We then followed the same experimental protocols in Table 2 to evaluate the prognostic prediction performance with these shuffled features. The performance is almost random as evaluated by the CI (around 50%). In addition, the permutation test exhibits a much larger variance in prediction results compared with the raw data in Table 2.

H Additional details on the use of Canopy and TUSV

We used Canopy with WES samples because it makes use of SNVs and CNAs and can make inferences from common VCF files. Canopy [13] infers subclones and predicts phylogenies based on an input VCF file specifically combining SNV and CNA data for inference, making it suitable for WES data. First, the clonal decomposition is explored by Markov chain Monte Carlo (MCMC) simulation, and assessed based on the maximum likelihood estimation (MLE). Users need to specify the range for the number of subclones to deconvolve, and also a range for the number of chains and the length of each chain for MCMC simulation. Bayesian information criterion (BIC) is used to determine the optimal number of clones in the specified range [14]. The clonal composition and the bifurcating tree with SNVs and CNAs on the edges are determined based on the posterior distribution. Canopy outputs the clonal compositions and phylogenetic trees, where each SNV or CNA is assigned to a specific edge, which are then extracted as phylogenetic features.

The tumor phylogenetic reconstruction tool TUSV [15] was used with WGS data. It optimizes for a minimum evolution CNA model assuming SVs accumulate via a perfect phylogeny. TUSV makes use of a coordinate descent algorithm to optimize the objective function, where the inner steps of coordinate descent are implemented through mixed integer linear programming (MILP). The MILP model implements a trade-off between the likelihood of CNAs described in observed breakpoints and the evolutionary cost of the phylogenetic tree. We heuristically extended the previously published TUSV for the present work to further incorporate SNVs through a simplified mutation model that excludes the possibility of recurrent mutation but allows for the loss of SNVs through allelic loss. In doing so, we are able to apply the existing validated TUSV code to joint SNV/SV/CNA data without modification by applying the same model constraints to SNVs as the published TUSV applied to SVs. The output of the extended TUSV is a set of inferred clones characterized by the subset of variations inferred in each and a phylogeny connecting those clones. We refer interested readers to the TUSV paper [15] for the quality assessment and case study of the inferred phylogenetic trees, and statistics of features extracted from the trees.

I Feature extraction and preprocessing

We have described the extraction of evolutionary features in the main text. Below are the details of extraction and preprocessing of other features.

Driver features The potential drivers of BRCA and LUCA came from two sources. First, we used the IntOGen database [1], where the top 20 drivers based on mutation counts in samples of each cancer type were collected. Second, we used the COSMIC database [2], where 20 common drivers were collected. See Table I in S1 Text for the full list of potential drivers of both BRCA and LUCA. We counted the times that a driver was perturbed by SNVs, indels, CNAs or SVs. Examples of common drivers are *TP53*, *PIK3CA*, and *GATA3*.

Clinical features All the clinical data for the TCGA samples were extracted from TCGA-reported clinical metadata downloaded from GDC. The clinical features of ICGC/PCAWG samples also came from GDC, as the samples sequenced using WGS are a subset of the TCGA samples. We collected the clinical feature set which provides a consensus representation of information likely to be available to clinicians at the time of diagnosis. We then removed the features that are available for fewer than half of the samples (Table J in S1 Text). We note that a large portion of these clinical features are in common between BRCA and LUCA. Examples of preserved clinical features are *person neoplasm cancer status*, *pathologic stage*, and cancer subtype (*histological type*).

Feature engineering We preprocessed, encoded, and imputed the clinical features according to their value types. For continuous values, missing values were filled with the median value of the cohort. For binary values, missing values were filled with the mode of the cohort, and the features were encoded by 0/1. For non-binary categorical values, missing values were filled with the mode, and a feature of k categories was mapped into k mutually exclusive binary features. We removed the category that appears least frequently to avoid collinearity. We made $\log_2(x+1)$ transformation to the features that have long-tail distributions, including driver, cumulative evolutionary, and most of the phylogenetic evolutionary features, so that the resulting feature distribution is close to a normal distribution. We removed the sparse clinical and genomic features that are non-zero in fewer than 5% samples. Finally, we mapped all features into the interval $[0,1]$ linearly.

Cumulative Feature	Definition	Included	
		WES	WGS
$T \to A$	mutation rates	✓	✓
$T \to G$	mutation rates	✓	✓
$T \to C$	mutation rates	✓	✓
$C \to A$	mutation rates	✓	✓
$C \to T$	mutation rates	✓	✓
$C \to G$	mutation rates	✓	✓
$N_l N_x N_r \to N_l N_y N_r$	trinucleotide mutation rates (96 in total)	✓	✓
snv_rate	total SNV rates	✓	✓
cna_rate	total CNA rates	✓	✓
cna_amp_rate	CNA duplication rates	✓	✓
cna_del_rate	CNA deletion rates	✓	✓
cna_lg_rate	rates of CNA above 500,000 nt	✓	✓
cna_sm_rate	rates of CNA below 500,000 nt	✓	✓
sv_rate	total SV rates		✓

Table G. List of cumulative evolutionary features. The mutation rates related to SNVs, CNAs and SVs of samples are included. All cumulative evolutionary features are in continuous value. We have 6 mutation rates and 96 trinucleotide mutation rates as features in total.

Phylogenetic Feature	Definition	Included	
		WES	WGS
num_clone	clone number	✓	✓
diversity	diversity of clone proportions	✓	✓
lg_clone_proportion	proportion of the largest clone	✓	✓
lg_clone_snv	SNV rates in the largest clone	✓	
lg_clone_cna	CNA rates in the largest clone	✓	✓
lg_clone_sv	SV rates in the largest clone		✓
height_topology	topological height of phylogeny	✓	✓
height	height of phylogeny	✓	✓
height_cna	height of phylogeny in unit of CNA rates		✓
height_sv	height of phylogeny in unit of SV rates		✓
branch_num	number of edges in phylogeny	✓	✓
branch_len	total edge lengths	✓	✓
branch_mean	average of edge lengths	✓	✓
branch_mean_cna	average of edge lengths in unit of CNA rates		✓
branch_mean_sv	average of edge lengths in unit of SV rates		✓
branch_max	maximum edge length	✓	✓
branch_max_cna	maximum edge length in unit of CNA rates		✓
branch_max_sv	maximum edge length in unit of SV rates		✓
branch_var	variance of edge lengths	✓	✓
branch_var_cna	variance of edge lengths in unit of CNA rates		✓
branch_var_sv	variance of edge lengths in unit of SV rates		✓

Table H. List of phylogenetic evolutionary features. Due to the different output of Canopy (phylogenetic model for WES) and TUSV (phylogenetic model for WGS), the sets of phylogenetic features are slightly different. The WGS data contain additional features related to CNA and SV rates.

Driver Feature	Included	
	BRCA	LUCA
TP53, PIK3CA, GATA3, MLL3, CDH1, NCOR1, MAP2K4, PTEN, AKT1, RUNX1, NF1, RB1, ARID1A, TBX3, MLL2, SPEN, LRP1B, ESR1, KMT2C, KMT2D, FOXA1, ERBB2	✓	✓
MAP3K1, MACF1, MED12, ATM, AKAP9	✓	
KEAP1, CDKN2A, KRAS, EGFR, STK11, KDR, FAT1, SVEP1, NFE2L2, FN1, NOTCH1, MLL		✓

Table I. List of driver features. The potential drivers come from both IntOGen and COSMIC databases. BRCA and LUCA share a large portion of drivers. We count the somatic mutation rates of both SNVs, indels, CNAs, and SVs in all drivers as the driver features. These features are in continuous value.

Clinical Feature	Data Type	Included	
		BRCA	LUCA
person neoplasm cancer status	binary	✓	✓
gender	binary	✓	✓
history of neoadjuvant treatment	binary	✓	✓
ethnicity	binary	✓	✓
pathologic stage	categorical	✓	✓
histological type	categorical	✓	✓
race	categorical	✓	✓
age at initial pathologic diagnosis	continuous	✓	✓
lab procedure her2 neu in situ hybrid outcome type	binary	✓	
breast carcinoma progesterone receptor status	categorical	✓	
breast carcinoma estrogen receptor status	categorical	✓	
lab proc her2 neu immunohistochemistry receptor status	categorical	✓	
margin status	categorical	✓	
her2 immunohistochemistry level result	categorical	✓	
number of lymphnodes positive by he	continuous	✓	
cytokeratin immunohistochemistry staining method micrometastasis indicator	continuous	✓	
menopause status	categorical	✓	
her2 neu chromosome 17 signal ratio value	continuous	✓	
her2 immunohistochemistry level result	continuous	✓	
her2 erbb pos finding cell percent category	continuous	✓	
fluorescence in situ hybridization diagnostic procedure chromosome 17 signal result range	continuous	✓	
anatomic neoplasm subdivision	categorical		✓

Table J. List of clinical features. BRCA and LUCA samples share a large portion of similar clinical features. Three data types are available: binary, categorical and continuous. Cancer subtype is denoted as *histological type*.

J Experiment setup and evaluation protocol

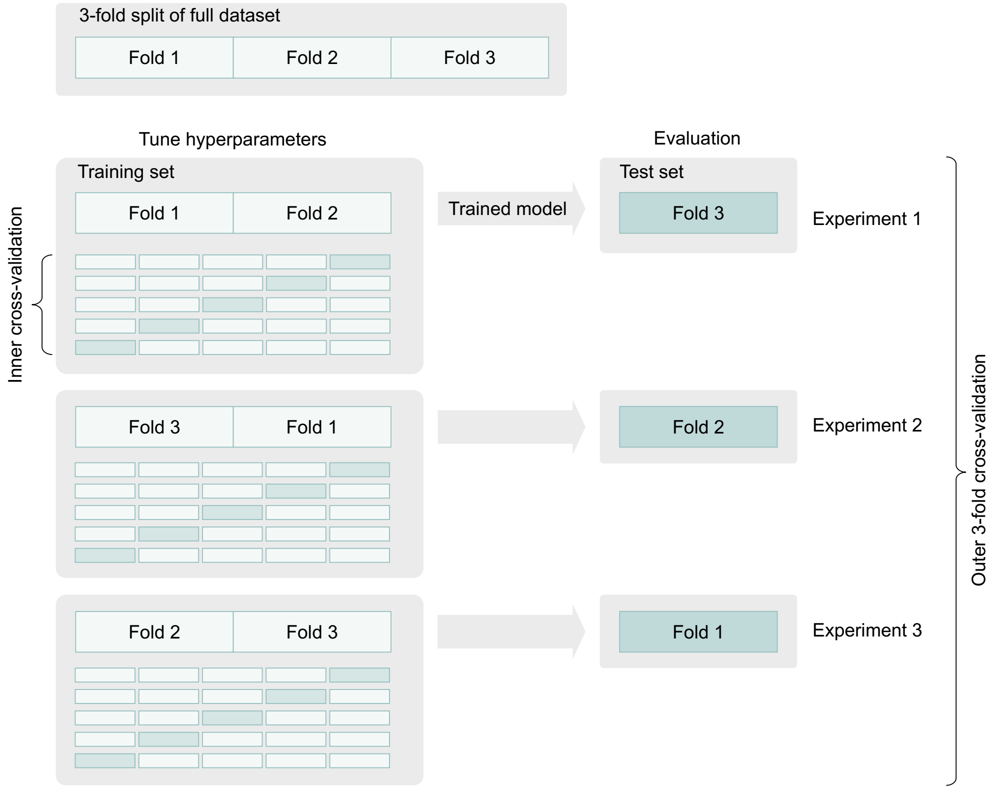

Fig H. Procedure of training, tuning, and unbiased evaluation through two-loop cross-validation. The whole dataset is split into three parts and evaluated through 3-fold outer cross-validation (CV) on the test sets. In each experiment of the outer CV, the ℓ_1- or ℓ_0-regularized model is tuned using an inner CV only on the training set. We used 3-fold inner CV for TCGA and leave-one-out inner CV (LOOCV) for ICGC throughout the work. We employed LOOCV for ICGC because it has a much smaller sample size. The utilization of two-loop CV prevents the problem of bias when we evaluate models with different complexities, e.g., Cox model using clinical features vs. Cox model using both clinical and genomic features, or ℓ_1-regularized model vs. ℓ_0-regularized model. In contrast, the model with larger complexity tends to perform "better" due to overfitting if using the single-loop CV.

References

1. Gonzalez-Perez A, et al. IntOGen-mutations identifies cancer drivers across tumor types. Nature Methods. 2013;10(11):1081.

2. Tate JG, et al. COSMIC: the catalogue of somatic mutations in cancer. Nucleic Acids Research. 2018;47(D1):D941–D947.

3. Liu CX, Li Y, Obermoeller-McCormick LM, Schwartz AL, Bu G. The Putative Tumor Suppressor LRP1B, a Novel Member of the Low Density Lipoprotein (LDL) Receptor Family, Exhibits Both Overlapping and Distinct Properties with

the LDL Receptor-related Protein. The Journal of Biological Chemistry. 2001;276:28889–28896.

4. Amin MB, et al. The Eighth Edition AJCC Cancer Staging Manual: Continuing to build a bridge from a population-based to a more “personalized” approach to cancer staging. CA: A Cancer Journal for Clinicians. 2017;67(2):93–99.

5. Tan KS, Eguchi T, Adusumilli PS. Competing risks and cancer-specific mortality: why it matters. Oncotarget. 2017;9(7):7272–7273.

6. Lyman GH, et al. Age and the Risk of Breast Cancer Recurrence. Cancer Control. 1996;3(5):421–427.

7. Elledge RM, et al. Estrogen receptor (ER) and progesterone receptor (PgR), by ligand-binding assay compared with ER, PgR and pS2, by immuno-histochemistry in predicting response to tamoxifen in metastatic breast cancer: A Southwest Oncology Group study. International Journal of Cancer. 2000;89(2):111–117.

8. Yang J, et al. The value of positive lymph nodes ratio combined with negative lymph node count in prediction of breast cancer survival. Journal of Thoracic Disease. 2017;9(6):1531.

9. Cserni G, et al. The value of cytokeratin immunohistochemistry in the evaluation of axillary sentinel lymph nodes in patients with lobular breast carcinoma. Journal of Clinical Pathology. 2006;59(5):518–522.

10. Liu H. Application of immunohistochemistry in breast pathology: a review and update. Archives of Pathology & Laboratory Medicine. 2014;138(12):1629–1642.

11. Sahmoun AE, Case LD, Santoro TJ, Schwartz GG. Anatomical distribution of small cell lung cancer: effects of lobe and gender on brain metastasis and survival. Anticancer Research. 2005;25(2A):1101–1108.

12. Loeb LA. A mutator phenotype in cancer. Cancer Research. 2001;61(8):3230–3239.

13. Jiang Y, Qiu Y, Minn AJ, Zhang NR. Assessing intratumor heterogeneity and tracking longitudinal and spatial clonal evolutionary history by next-generation sequencing. Proceedings of the National Academy of Sciences. 2016;113(37):E5528–E5537.

14. Schwarz G. Estimating the Dimension of a Model. Annals of Statistics. 1978;6(2):461–464.

15. Eaton J, Wang J, Schwartz R. Deconvolution and phylogeny inference of structural variations in tumor genomic samples. Bioinformatics. 2018;34(13):i357–i365.
